# Magnetotactic bacteria affiliated with diverse *Pseudomonadota* families biomineralize intracellular Ca-carbonate

**DOI:** 10.1093/ismejo/wrae260

**Published:** 2025-01-07

**Authors:** Camille C Mangin, Karim Benzerara, Marine Bergot, Nicolas Menguy, Béatrice Alonso, Stéphanie Fouteau, Raphaël Méheust, Daniel M Chevrier, Christian Godon, Elsa Turrini, Neha Mehta, Arnaud Duverger, Cynthia Travert, Vincent Busigny, Elodie Duprat, Romain Bolzoni, Corinne Cruaud, Eric Viollier, Didier Jézéquel, David Vallenet, Christopher T Lefèvre, Caroline L Monteil

**Affiliations:** Aix-Marseille Université, CNRS, CEA, BIAM, UMR7265 Institut de Biosciences and Biotechnologies d’Aix-Marseille, Cadarache research centre, F-13115 Saint-Paul-lez-Durance, France; Sorbonne Université, Muséum National d’Histoire Naturelle, UMR CNRS 7590, Institut de Minéralogie, de Physique des Matériaux et de Cosmochimie (IMPMC), 4 Place Jussieu, 75005 Paris, France; Aix-Marseille Université, CNRS, CEA, BIAM, UMR7265 Institut de Biosciences and Biotechnologies d’Aix-Marseille, Cadarache research centre, F-13115 Saint-Paul-lez-Durance, France; Sorbonne Université, Muséum National d’Histoire Naturelle, UMR CNRS 7590, Institut de Minéralogie, de Physique des Matériaux et de Cosmochimie (IMPMC), 4 Place Jussieu, 75005 Paris, France; Aix-Marseille Université, CNRS, CEA, BIAM, UMR7265 Institut de Biosciences and Biotechnologies d’Aix-Marseille, Cadarache research centre, F-13115 Saint-Paul-lez-Durance, France; Génomique Métabolique, Genoscope, Institut François Jacob, CEA, CNRS, Univ Evry, Université Paris-Saclay, 91057 Evry, France; Génomique Métabolique, Genoscope, Institut François Jacob, CEA, CNRS, Univ Evry, Université Paris-Saclay, 91057 Evry, France; Aix-Marseille Université, CNRS, CEA, BIAM, UMR7265 Institut de Biosciences and Biotechnologies d’Aix-Marseille, Cadarache research centre, F-13115 Saint-Paul-lez-Durance, France; Aix-Marseille Université, CNRS, CEA, BIAM, UMR7265 Institut de Biosciences and Biotechnologies d’Aix-Marseille, Cadarache research centre, F-13115 Saint-Paul-lez-Durance, France; Aix-Marseille Université, CNRS, CEA, BIAM, UMR7265 Institut de Biosciences and Biotechnologies d’Aix-Marseille, Cadarache research centre, F-13115 Saint-Paul-lez-Durance, France; Sorbonne Université, Muséum National d’Histoire Naturelle, UMR CNRS 7590, Institut de Minéralogie, de Physique des Matériaux et de Cosmochimie (IMPMC), 4 Place Jussieu, 75005 Paris, France; Sorbonne Université, Muséum National d’Histoire Naturelle, UMR CNRS 7590, Institut de Minéralogie, de Physique des Matériaux et de Cosmochimie (IMPMC), 4 Place Jussieu, 75005 Paris, France; Sorbonne Université, Muséum National d’Histoire Naturelle, UMR CNRS 7590, Institut de Minéralogie, de Physique des Matériaux et de Cosmochimie (IMPMC), 4 Place Jussieu, 75005 Paris, France; Université Paris Cité, Institut de Physique du Globe de Paris, CNRS, Paris F-75005, France; Sorbonne Université, Muséum National d’Histoire Naturelle, UMR CNRS 7590, Institut de Minéralogie, de Physique des Matériaux et de Cosmochimie (IMPMC), 4 Place Jussieu, 75005 Paris, France; Aix-Marseille Université, CNRS, CEA, BIAM, UMR7265 Institut de Biosciences and Biotechnologies d’Aix-Marseille, Cadarache research centre, F-13115 Saint-Paul-lez-Durance, France; Sorbonne Université, Muséum National d’Histoire Naturelle, UMR CNRS 7590, Institut de Minéralogie, de Physique des Matériaux et de Cosmochimie (IMPMC), 4 Place Jussieu, 75005 Paris, France; Genoscope, Institut François Jacob, CEA, CNRS, Université Évry, Université Paris-Saclay, 91057 Evry, France; Laboratoire des Sciences du Climat et de l’Environnement, LSCE–IPSL, CEA–CNRS–UVSQ–Université Paris-Saclay, 91198, Gif-sur-Yvette, France; Université Paris Cité, Institut de Physique du Globe de Paris, CNRS, Paris F-75005, France; UMR CARRTEL, INRAE & Université Savoie Mont Blanc, Thonon-les-Bains 74200, France; Génomique Métabolique, Genoscope, Institut François Jacob, CEA, CNRS, Univ Evry, Université Paris-Saclay, 91057 Evry, France; Aix-Marseille Université, CNRS, CEA, BIAM, UMR7265 Institut de Biosciences and Biotechnologies d’Aix-Marseille, Cadarache research centre, F-13115 Saint-Paul-lez-Durance, France; Aix-Marseille Université, CNRS, CEA, BIAM, UMR7265 Institut de Biosciences and Biotechnologies d’Aix-Marseille, Cadarache research centre, F-13115 Saint-Paul-lez-Durance, France

**Keywords:** environmental microbiology, biomineralization, magnetotactic bacteria, carbonatogenesis, calcium carbonate

## Abstract

Intracellular calcium carbonate formation has long been associated with a single genus of giant *Gammaproteobacteria, Achromatium.* However, this biomineralization has recently received increasing attention after being observed in photosynthetic *Cyanobacteriota* and in two families of magnetotactic bacteria affiliated with the *Alphaproteobacteria.* In the latter group, bacteria form not only intracellular amorphous calcium carbonates into large inclusions that are refringent under the light microscope, but also intracellular ferrimagnetic crystals into organelles called magnetosomes. Here new observations suggest that magnetotactic bacteria previously identified in the sediments and water column of Lake Pavin (France) were only a small fraction of the diversity of bacteria producing intracellular amorphous calcium carbonates. To explore this diversity further, we conducted a comprehensive investigation of magnetotactic populations with refractive granules using a combination of environmental microbiology, genomic and mineralogy approaches on cells sorted by micromanipulation. Several species belonging to divergent genera of two *Pseudomonadota* classes were identified and characterized. Scanning transmission electron microscopy coupled with energy-dispersive X-ray spectrometry support that all these species indeed form intracellular amorphous calcium carbonates. Cryo soft X-ray tomography experiments conducted on ice-vitrified cells, enabled 3D investigation of inclusions volume, which was found to occupy 44–68% of the cell volume. Metabolic network modeling highlighted different metabolic abilities of *Alpha*- and *Gammaproteobacteria,* including methylotrophy and CO_2_ fixation *via* the reverse Krebs cycle or the Calvin-Benson-Bassham cycle. Overall, this study strengthens a convergent evolution scenario for intracellular carbonatogenesis in *Bacteria*, and further supports that it is promoted by the fixation of CO_2_ in anoxic environments.

## Introduction

Due to their highly diversified metabolic functions and abundance, microorganisms are important drivers of the global ecosystem functioning through their interactions with their biotic and abiotic environment [1]. Their metabolic activity can affect and be affected by the bioavailability of essential basic elements (i.e. C, H, N, O, P, S) and many alkaline earth or transition metals, on which they depend for growth, activity, or survival [2]. To respond to environmental fluctuations, microorganisms have developed strategies for controlling their needs and element homeostasis, including the uptake, storage, and utilization. Some of them rely on the ability to form mineral phases within their cells, periplasm or extracellular space through a process known as biomineralization [3]. This process is ubiquitous in nature and numerous microbial species synthesize a great diversity of biominerals varying widely in chemical composition and structure [4]. However, we still know little about the biodiversity and evolution of biomineralizing microorganisms, the mechanisms of mineral formation, and the role of minerals in life adaptation and diversification.

Most of the biominerals formed by prokaryotes are extracellular and are secondary byproducts of their metabolic activities that create a local chemical environment favoring mineral precipitation [4, 5]. However, there are few, less documented cases where this formation is intracellular, genetically regulated and controlled (e.g. by the production of proteins capable of nucleating and growing minerals). This results in the formation of dedicated inclusions isolating the mineral from the cytoplasm. Microbial intracellular biominerals may be composed of silica, sulphates, sulfur, phosphates, oxides and carbonates associated with different alkaline earth elements or transition metals [6–8]. The most emblematic case of controlled intracellular biomineralization is that achieved by magnetotactic bacteria (MTB) [9]. These aquatic bacteria produce magnetosomes, i.e. nano-sized organelles composed of ferrimagnetic crystals surrounded by a lipid bilayer [10]. Magnetosomes can be composed of magnetite (Fe_3_O_4_) or greigite (Fe_3_S_4_) and provide a magnetic moment to the cell. Together with a chemo-aerotactism, they assist bacteria in their motility by magnetotaxis and guide them along magnetic field lines [11, 12]. This group of bacteria is distributed in aquatic and sedimentary environments worldwide and are usually detected in stratified environments below and near the oxic-anoxic transition zone [13, 14]. Different MTB can form chains of magnetosomes different in size, number, shape, organization or chemical composition [15]. However, a species generally forms only one type of magnetosome chain in the same redox conditions. For example, magnetotactic sulphate-reducing bacteria form bullet-shaped magnetosomes of magnetite, whereas *Alphaproteobacteria* form cuboctahedral or prismatic magnetite magnetosomes. The underlying genetic determinism, molecular machinery, ecological role and evolution of magnetosome formation have been the subject of interdisciplinary research. This process represents the most documented case of biomineralization in prokaryotes [10].

The polyphyletic distribution of MTB [16], the diversity of their metabolic activities [17], and the possibility to selectively sort MTB from complex ecosystems using magnets make MTB great models to study intracellular minerals. This last decade, several studies have highlighted the propensity of MTB to form additional intracellular compartments concentrating diverse chemical elements. For example, some magnetotactic *Magnetococcaceae* and *Azospirillaceae* are also known to form elemental sulfur (S^0^) and polyphosphate globules with varying phosphorus, calcium, magnesium and potassium contents [18–20]. In 2014, both magnetosomes and large calcium-enriched granules were observed in an uncultured giant (~13 × 8 μm) rod-shaped *Gammaproteobacteria* named GRS-1, isolated from a freshwater pond in Kanazawa (Japan) [21]. In 2022, the first documented cases of silicification and periplasmic copper sulfide biomineralization were reported in deep-branching magnetotactic bacteria [22] and in a greigite producer [23], respectively. Recently, another case of double biomineralization was observed in two *Alphaproteobacteria* families capable not only of making magnetosomes, but also calcium carbonate inclusions of varying sizes: (i) a magnetotactic spirillum, XQGS-1, isolated from the freshwater sediments of Xingqinggong Lake (Xi’an City, Shaanxi Province, China) [24] and (ii), a magnetotactic slightly-curved rod, CCP1, in the sediments and the water column of a meromictic lake (Lake Pavin, Auvergne, France) [25]. The bacterium XQGS-1 forms 2 to 3 granules of poorly crystalline calcium carbonate per cell, each measuring ~100 nm in width. By contrast, CCP1 cells contain 2 or 4 inclusions of amorphous calcium carbonate (iACC), which are highly refractive under the light microscope and measure ~1 μm in width and occupy most of the intracellular volume. Overall, the coexistence of ferrimagnetic biomineralization and intracellular carbonatogenesis in these MTB (_iACC_MTB) seems to be more widespread than previously thought and has raised several questions about intracellular carbonatogenesis, as this biomineralization is much less documented.

Before the discovery of _iACC_MTB, this type of biomineralization was described in two major bacterial phyla only. The first iACC-forming bacterium (_iACC_B) ever observed is *Achromatium oxaliferum*, a giant (up to ~130 x 50 μm) uncultured sulfur-oxidizing *Gammaproteobacteria* [26]. Cells contain large inclusions (up to 1.5 μm in length) occupying up to 77% of the cell volume [27]. After its discovery by W. Schewiakof in 1893, *Achromatium* was observed at oxic-anoxic boundaries of a wide variety of freshwater and marine environments worldwide [26–28]. More than a century later, Couradeau *et al*. [29] isolated a new _iACC_B species, *Gloeomargarita lithophora*, from stromatolites collected in a Mexican crater lake*.* This member of the *Cyanobacteriota* (formerly *Cyanobacteria*), contains iACC measuring around 270 nanometers in size, composed of calcium, magnesium, strontium and barium and occupying up to 8% of the cell volume. Later, a large screening of cultivated species revealed that carbonatogenesis was actually widespread in *Cyanobacteriota* and ubiquitous in oxygenated freshwater and marine waters [30]. For all _iACC_B groups, including _iACC_MTB, carbonatogenesis can occur in solutions under-saturated with respect to all CaCO_3_ mineral phases, suggesting some energy expense, possibly in relation with the active transport of alkaline earth elements and/or C into vesicles [31]. However, the metabolic pathways and genetic determinants associated with iACC formation are still poorly understood [25, 26].

Non-magnetotactic _iACC_B can fix CO_2_ through the Calvin-Benson-Bassham (CBB) cycle via the ribulose-1,5-bisphosphate carboxylase-oxygenase (RuBisCO) [7, 32], which could favor intracellular carbonate formation [33]. Additionally, it has been suggested that some *Achromatium* populations may also use the reverse citric acid cycle (rTCA, or reverse Krebs cycle) [34]. It is not clear yet whether iACC formation has the same molecular origin in *Gammaproteobacteria* and *Cyanobacteriota*. A gene, *ccyA*, member of a new protein family called calcyanin, has been associated with iACC biomineralization in *Cyanobacteriota* [35]. However, this gene has not been detected in *Achromatium* genomes yet, and no similar comparative genomics study has ever proposed alternative candidate genes to iACC formation in this genus. Conversely, genes coding for P-type Ca^2+^ transporters were suggested to play a role in iACC biomineralization by *Achromatium,* but were not systematically found in cyanobacterial genomes [36]. Last, _iACC_B belonging to the *Cyanobacteriota* and *Gammaproteobacteria* have very contrasted ecological niches: *Cyanobacteriota* species are photosynthetic, whereas *Achromatium* species are sulfur-oxidizers in reduced, possibly aphotic aquatic habitats. For the _iACC_MTB, metabolic potentials and functional roles are still unknown as no good quality genome assembly has been obtained to date. But, like for the two other models, it can be hypothesized that iACC could: 1) serve as a source of remobilizable inorganic C for the organism; 2) buffer pH variations induced by the oxidation of H_2_S to sulfate in *Achromatium* or photosynthesis in *Cyanobacteriota* [6]; or 3) serve as ballasts for cells owing to their density (> 2 g.cm^−3^) [28, 29]. These hypotheses are still the subject of debate as data are still limited by the lack of cultures for several _iACC_B models. In-depth characterization of diversity through culture-independent approaches can help to better understand the biology, ecology and evolution of this group of microorganisms.

Here, several observations in the sediments of a meromictic lake (Lake Pavin, Auvergne, France) suggested that magnetotactic calcifying microorganisms could be more diverse than originally thought [25]. Indeed, bacteria with refractive granules of different sizes, numbers and organizations were regularly observed among magnetotactic populations inhabiting the sediments. As the refringence may indicate the presence of carbonates, we conducted a deeper characterization of these MTB via a combination of environmental microbiology, genomic and mineralogy approaches on cells sorted by micromanipulation. We first classified them into different morphotypes based on their morphology and ultrastructure. In parallel, we validated the chemical composition of their inclusions and magnetosomes. Finally, we sequenced representative genomes for each morphotype to investigate their identity and metabolic potential. This study characterized magnetotactic bacteria affiliated with several *Pseudomonadota* families and genera that biomineralize intracellular amorphous calcium carbonate with distinctive ultrastructural traits for each genomic group. Although some of them resemble to the non-magnetotatic calcifying *Achromatium*, _iACC_MTB are represented by different genetic groups. As for magnetosome formation, iACC inclusions formation is likely triggered by specific genetic determinants and appears as a polyphyletic trait associated with several metabolic pathways, including methylotrophy and autotrophy. We discuss the possible scenarios that might have led to this diversity pattern and propose different hypotheses for their biogeochemical niches and role in inorganic carbon (iC) sequestration.

## Materials and methods

### Site description and sample collection

The diversity of _iACC_MTB was assessed in both the sediments and the water column of Lake Pavin, located in the Massif Central (France, Auvergne) at an altitude of 1197 m (45°29′41′ N, 002°53′12″E). Lake Pavin is a volcanic crater lake formed by a phreato-magmatic eruption which resulted from the encounter of a lava rise and a water table. This event is estimated to have taken place approximately 6900 to 7000 years ago [37]. The lake is circular in shape, has a diameter of 750 m and a maximum depth of 92 m. It is meromictic, i.e. permanently chemically stratified [38, 39]. There are two major compartments: (i) the monimolimnion between ~60 and 92 m depth, in which water permanently remains anoxic, and (ii) the mixolimnion encompassing surface waters down to ~60 m in depth, stirred annually. The oxic-anoxic transition zone, in the mesolimnion, lies between these two compartments and experiences intermediate redox conditions with strong chemical gradients. This zone can extend over several meters and its location varies from year to year between 50 and 60 m in depth [40]. MTB are generally found just below in the anoxic layers [20].

Samples of both the sediments and the water column were collected over several campaigns between 2015 and 2023. For the sediments, samples from the shore were collected at three locations as previously described [25] (see details in Fig. S1). Several one-liter glass bottles were filled with 400–500 ml of surface sediment (~ 5 cm deep) and then filled with the local water. Mesocosms were stored on a laboratory bench at ambient temperatures (∼20°C) before processing. The abundance and diversity of MTB populations in the mesocosms were regularly checked over time. In some of them, MTB populations were observed several years after the sediment sampling. Water column samples were collected at depths between 45 and 65 m across and beneath the oxic-anoxic transition zone using a pumping system which enables to collect water at a precise depth [40], thereby increasing both sampling speed and vertical resolution (i.e. with a 10 cm precision). Additional details are given in Supplementary Notes: Method S1 and 2. Unlike MTB in the sediments, MTB in water samples can be preserved only for a couple of days when stored at 4°C, possibly due to the chemical gradient disappearance.

### Magnetic enrichment and light microscopy observations

Magnetic enrichment was performed as previously described [25]. The process involved concentrating environmental MTB and separating them from other non-magnetic microorganisms using a magnet, with the south pole positioned next to the sample bottles. For MTB harvested from the sediment, the magnet was placed above the sediment–water interface, and for MTB harvested from the water column, it was positioned at the center. This procedure was carried out for 1–4 h. Observations of the magnetically concentrated cells were performed using the hanging drop technique [41] under a Zeiss Primo Star optical microscope equipped with phase contrast and differential interference contrast optics. Motility and magnetotactic behavior were also observed and recorded under a Leica LMD6000 light microscope equipped with a Leica DMC 4500 camera (Leica Microsystems GmbH, Germany). The _iACC_MTB have the particularity of forming very bright and refractive granules, visible under the light microscope. This distinctive feature was used to classify morphotypes based on light microscopy observations prior an in-depth characterization by a single-cell approach.

### Cell sorting and whole genome amplification

In this approach, magnetically concentrated MTB were sorted using an InjectMan® NI2 micromanipulator and a CellTram® vario manual hydraulic microinjector (Eppendorf) mounted on a Leica DM IL LED microscope. The microscope and micromanipulator were placed inside a chamber, washed with 70% ethanol and sterilized by 2 h germicidal ultraviolet irradiation (λ = 254 nm) before use. A 10-μl drop containing magnetically concentrated cells was gently added to a 20-μl drop of filtered (0.2 μm) water from Lake Pavin on a hydrophobic coverslip to magnetically transfer the magnetotactic cells to the clean filtered water. Micromanipulation involves the precise aspiration and expulsion of individual cells using a microinjector and a sterile microcapillary (TransferTip® (ES) with a 4 or 15-μm internal diameter) either (i) onto a transmission electron microscopy grid for further ultrastructural characterization or (ii), into a drop of 4 μl of phosphate-buffered saline solution, further stored at −20°C prior to genome amplification.

Whole genome amplification was performed on sorted single cells to obtain sufficient genomic DNA for 16S rRNA gene and genome sequencing. We used the multiple displacement amplification (MDA) technique and the REPLI-g Single Cell kit (QIAGEN) following the manufacturer’s instructions. Double-stranded gDNA concentration was measured using a QuBitTM 4 fluorometer (ThermoFisher Scientific). At least three and up to 10 cells of each morphotype were sorted by micromanipulation and their genomes were independently amplified.

### Cloning and sequencing of 16S rRNA gene

The 16S rRNA gene sequence was amplified with the 27f and 1492r universal primers [42], cloned and sequenced as previously described [25]. All 16S rRNA gene sequences with an identity threshold strictly higher than 97% were grouped into an operational taxonomic unit (OTU). Sequences of the different OTUs were compared using the Basic Local Alignment Search Tool (BLAST) with those in the public NCBI “Nucleotide Collection (Nr / Nt)” database and the SILVA SSU 138.2 database (November 2023), first selecting sequences from type strains only, and a second time including those from all environmental and/or uncultivated sequences. Sequences aligned over > 95% of their length with the best alignment score and percentage identity were further used for phylogenetics.

### Fluorescence *in situ* hybridization

Each 16S rRNA gene sequence obtained by PCR or from genome assembly, was validated by Fluorescence *in situ* hybridization (FISH). Sequence complementarity is shown by the fluorescence at specific wavelengths. The FISH protocol used in this study is similar to that described in previous studies [25, 43]. An ATTO488-labelled probe was designed for each morphotype using alignments of the 16S rRNA gene sequences from the three different bacteria on which FISH was performed and the most similar public sequences retrieved after a BLAST search. Probe specificity was evaluated by using the PROBE_MATCH program in the RDP-II [44] and was further validated by showing that the probes do not bind to other MTB than the targeted _iACC_MTB on the same slide preparations for confocal microscopy observations. A complete description of the protocol and probes is given in Supplementary Notes: Method S3.

### Transmission electron microscopy

A 2-μl drop of magnetically concentrated MTB cells were deposited onto the Transmission electron microscopy (TEM) grids coated with a carbon film and poly-L-lysine to enhance cell adhesion and passive adsorption. Thanks to their magnetotactic abilities, cells were attracted to the edge of the drop over a 15-minutes period by a magnet positioned near the appropriate side if the grid. The grids were then rinsed with filtered milliQ water. Some ultrathin (~100 nm in thickness) sections of bacterial pellets were also prepared by ultramicrotomy (Supplementary Notes: Method S4) and stained with uranyless and 3% lead citrate (Reynolds Lead Citrate, Uranyless). Electron microscopy analyses were performed with a Tecnai G2 BioTWIN (FEI Company) microscope equipped with a charge-coupled-device (CCD) camera (Olympus Soft imaging Solutions GmbH) using an accelerating voltage of 100 kV.

High-resolution transmission microscopy (HRTEM), z-contrast imaging in the high-angle annular dark field (STEM-HAADF) mode, and elemental mapping by X-ray energy-dispersive spectrometry (XEDS) were carried out using a JEOL 2100 F microscope. This machine, operating at 200 kV, was equipped with a Schottky emission gun, an ultrahigh resolution pole piece, and an ultrathin window JEOL XEDS detector. HRTEM images were obtained with a Gatan US4000 CCD camera and a JEOL XEDS ultrafine window detector (i.e. Si(Li) detector). The intensity and size of the beam used in STEM-XEDS mode were optimized to maintain good spatial resolution and limit beam damages to the inclusions. The same applies to the dwell-time (counting time for each pixel).

### Scanning electron microscopy

Cell pellets or single cells were transferred onto a polycarbonate filter with 0.2 μm pores arranged in a Swinnex support system. This filter was then rinsed with milliQ water, dried, and attached to an aluminum scanning electron microscopy (SEM) stub using double-sided carbon tape. Samples were coated with evaporated carbon so that the entire surface became conductive to allow the flow of excess electrons. Analyses were performed using a Zeiss Ultra55 SEM (Carl Zeiss AG, Germany). The accelerating voltage power was set to 10–15 keV and samples were placed at a working distance of 7.3–7.6 mm for imaging and chemical analyses by XEDS.

### Cryo soft X-ray tomography

Imaging was conducted at ALBA synchrotron using cryo transmission X-ray microscopy at Mistral beamline (Barcelona, Spain) [45]. Five microliters of the magnetically-concentrated MTB and 1 μl of a suspension of 100-nm gold nanoparticles (BBI Solutions, 5X concentrated) were added to a poly-l-lysine-coated TEM grid (Quantafoil R2/2 holey carbon). Gold nanoparticles deposited on the grid served as fiducial markers for projection alignment prior to tomographic reconstruction. The grid was incubated horizontally for at least 1–2 min to allow deposition of bacteria onto the grid. The grid was then loaded into a Leica EM GP plunge freezer with 95% humidity chamber, blotted from the back with filter paper (3 s blotting time) and then dropped into a liquid ethane container (−180°C) cooled by liquid nitrogen. Grids were transferred to the MISTRAL beamline cryo chamber for measurement.

A tilt series of projections from −70° to +70° (range reduced by 5° for some samples) was collected every 1° with an incident X-ray energy of 520 eV. Exposure time was 2 s for each projection. A 40-nm Fresnel zone plate was used with an effective pixel size of 12 nm. The projections were normalized with the incoming flux and deconvolved with the measured point spread function of the optical system [46]. Alignment of projections was done with Etomo using Au nanoparticle fiducials of 100 nm. Tomographic reconstruction and simultaneous iterations reconstruction technique deconvolution were performed using IMOD. Volume segmentation, volume calculations and visualization of tomograms was conducted using Microscopy Image Browser [46].

### Statistical analyses

Images obtained using light and electron microscopy (TEM and SEM) were used to measure cells and magnetosomes size and shape factor (length / width), and iACC size (diameter) using the ImageJ software (v 1.53). Several cells were used to estimate an average and a standard deviation for each feature and morphotypes 1, 2, 3, 4 and 5 (including the one described previously [25]), using the R software v4.1.2 [47].

### Genome sequencing and assembly

The genomic DNA was sequenced at the Genoscope (Evry, France) using a combination of Illumina and Oxford Nanopore Technologies (see Supplementary Notes: Method S5 for complete procedure). Single-cell amplified genomes (SAG) were assembled using SPAdes software v3.13.0 with –k auto and –sc options). One SAG led to one non redundant, homogeneous and high-quality draft genome, which suggests that polyploidy is unlikely for _iACC_MTB to the contrary of what was observed for *A. oxaliferum* before [48].

In parallel to SAG sequencing, a metagenomic approach was employed to assemble the genome of the _iACC_MTB populations from the water column. The DNA was extracted from a pellet of 10^9^ MTB cells collected from 200 L of water in October 2021 (see details in Supplementary Notes: Method S1). Then total DNA was extracted with a conventional phenol/chloroform method [49]. An equivalent sequencing and assembly procedure was performed using the same technologies and assembler as for SAGs, but with –meta options of SPAdes [50].

### Genome binning, curation, and quality control

Assemblies were processed following the “Anvi’o User Tutorial for Metagenomic Workflow” to visualize them (https://merenlab.org/2016/06/22/anvio-tutorial-v2/) [51]. Briefly, contigs coverage values were calculated as the ratio of total length of mapped reads to the total length of the scaffold, using bowtie2 v2.4.2 [52] for mapping. Genes were predicted using prodigal v2.6.3 [53] and contig taxonomic assignment was performed using Kaiju v1.7.4 [54]. For each assembly, contigs longer than 1000 bp were visualized in Anvi’o version 6.2 [51] and genomes were identified interactively on the basis of tetra-nucleotide frequency, read coverage, GC content and taxonomic profile. The resulting SAG and metagenome-assembled-genomes (MAG) were manually curated using refineM v0.1.2 [55] to remove contigs with aberrant GC content, taxonomy and/or tetranucleotide frequencies. This approach created ~20 bins of varying quality, each corresponding to a SAG or a MAG. Assembly completeness and contamination of the different SAGs and MAGs were assessed using both checkM v1.0.11 [56] *via* the lineage-specific workflow, and Anvi’o using anvi-estimate-genome-completeness tool with the defaults HMM collections of single-copy core genes named Bacteria_71. Only bins of high quality (i.e. > 90% completeness, < 5% redundancy) were kept. When binning the metagenome assembly, the MAG having a 16S rRNA gene sequence and a taxonomic assignation identical to that of sorted cells was associated with the _iACC_MTB population of the water column.

### Genome-based taxonomic classification and molecular phylogeny

Phylogenetic trees were built from the whole genomes of the main morphotypes. First, GTDB-Tk v2.1.1 [57] was used to assign each SAG / MAG to a taxonomic group based on the GTDB classification [58]. Then, all high-quality genomes (i.e. > 90% complete with < 5% redundancy according to checkM values on GTDB) were downloaded from the Genbank database [59] in November 2023, and rapidly re-annotated with PROKKA v1.14.6 [60] to homogenize coding sequence predictions. The sequences of the 120 (bac120) phylogenetically informative markers [57], were then extracted using pyhmmer 0.8.0 [61] and the Hidden Markov Models (HMMs) of each of the 120 markers using the bit score gathering threshold for each profile (used to define a corresponding homologue). To limit the number of gaps due to incomplete assemblies, we selected only genomes in which more than 90% of markers were detected (except for the _iACC_MTB genomes and those of the closest MAG), and eliminated markers detected in less than 90% of genomes. Multiple sequence alignments (MSAs) were then performed with MAFFT v7.490 [62], trimmed with BMGE v1.12 [63] for each marker and concatenated into a single alignment. Trees were constructed using the maximum likelihood method implemented in the IQ-TREE v2.2 software [64, 65] and a substitution model was selected from the global alignment with ModelFinder [66] with the –MFP option. Branch robustness was estimated by the SH-aLRT likelihood ratio test (1000 replicas), and by the nonparametric bootstrap method (500 resamples). Trees were drawn and edited using FigTree v1.4.4 [67] and Inkscape software (Inkscape Project, 2020, https://inkscape.org). The average amino-acid identity (AAI) value was also used for genus delineation [68, 69].

### Functional annotation and metabolic network modeling

Metabolic pathways were inferred using the MetaCyc database [70], which offers an extensive catalog of metabolic pathways, enzymatic reactions, enzymes, chemical compounds, genes and review-level information for organisms in the three domains of life. This database includes 3105 pathways and 18 566 reactions in the version 27.0 that was used in this study. Metabolic pathway reconstruction was performed using the PathoLogic algorithm of Pathway Tools [71]. Beforehand, Prokka v 1.14.5 [60] was used to perform gene calling for each genome. Then, functional annotation was performed with KofamScan v.1.3.0 [72], that assigns KEGG Orthologs (KO) family numbers to protein sequences using HMMs database of KO and the pre-computed adaptive score thresholds. Next, we created Pathway Tools input files associating enzymatic activities to protein-coding genes thanks to their Enzyme Commission (EC) numbers or MetaCyc reaction identifiers when cross-references between KO, KEGG and MetaCyc identifiers were available. Finally, the obtained Pathway/Genome Databases (PGDB) were queried using the PythonCyc API v2.0.2 (https://github.com/networkbiolab/PythonCyc) to compute pathway completeness rates for each genome and generate a pathway/genome matrix. The genomes were also analyzed using the MicroScope platform [73] for further comparative analysis and expert functional annotation.

## Results

### High proportions of MTB inhabiting sediments and water column produce large refractive inclusions

**Figure 1:**
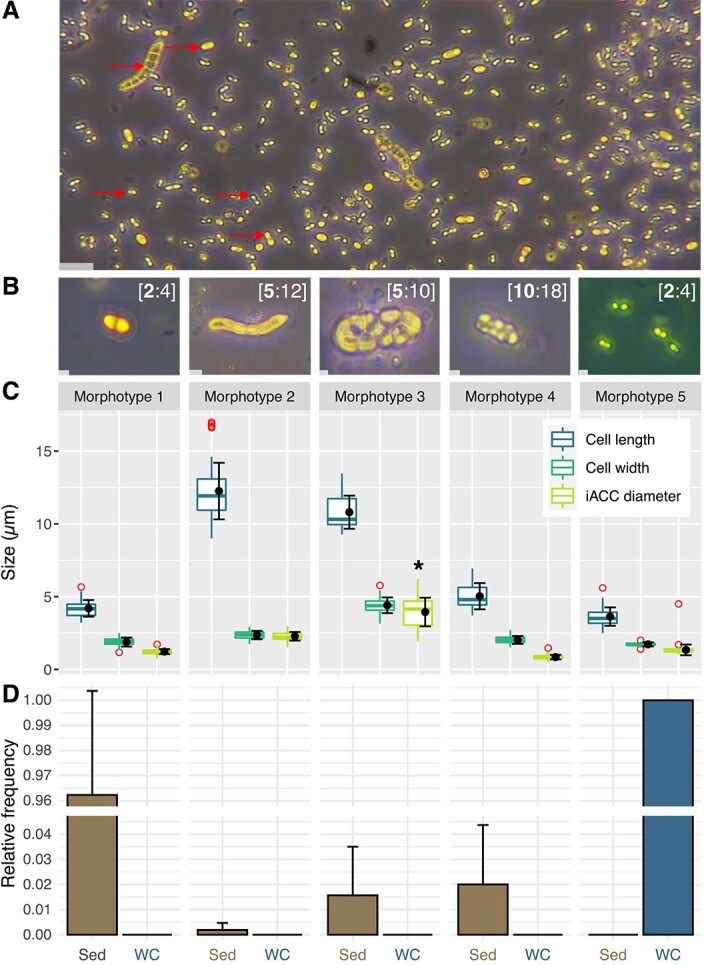
**Morphologies and relative frequencies of the five most abundant and regularly observed MTB populations with large refractive inclusions (**_**iACC**_ **MTB-*like*) in the shallow sediments and the water column of Lake Pavin.** (**A**) Representative light microscopy image of north-seeking MTB diversity in sediments, bearing highly refractive granules (yellow-colored structures such as those indicated by an arrow). The scale bar represents 9 μm. (**B**) Representative light microscopy images of the five morphotypes representing > 90% of _iACC_MTB-*like* in the Lake Pavin. Values in square brackets correspond to the range of inclusions numbers per cell from the lowest (bold value, representing most of the populations) to the highest value. Scales bars represent 1 μm. (**C**) Boxplots showing the distribution of the cell length, cell width and iACC inclusions diameter per morphotype. The iACC inclusion size could not be precisely measured for the morphotype 3 because inclusions shape and size were inconsistent. Only a rough estimation (marked with an *) could be made. Although similar, _iACC_MTB of the water column and those of the morphotype 1 in the sediment have different magnetosomes chains (Fig. 2C and D). Consequently, _iACC_MTB from the water column were classified as a distinct fifth morphotype. Measurements were performed from several cells: n = 33, 48, 20, 39, and 40 for morphotypes 1, 2, 3, 4, and 5, respectively. Estimations of iACC diameter were performed from 94, 107, 32, 103, and 90 inclusions, respectively. (**D**) Relative frequency of each _iACC_MTB-*like* morphotype in the sediments (Sed) and the chemocline of the water column (WC). Averages and standard deviations are plotted in black on both panels.

Cell pellets obtained from the magnetic concentration of microbial communities inhabiting the sediments and the water column were observed in a hanging-drop under the optical microscope. MTB can be identified by switching the polarity of a magnetic bar next to the slide: they swim parallel to the magnetic field whereas non-magnetic bacteria move randomly through the drop. In line with the first report of _iACC_MTB in Lake Pavin [25], a high cell density of north-seeking MTB (up to ~10^5–6^ cells/ml) was observed after 3 h of magnetic enrichment in all freshly collected sediment samples. MTB populations were represented by different morphologies including spirilla, cocci, vibrios, and rods, some of which contained large, highly refractive inclusions under the light of the optical microscope (Fig. 1A). Among these MTB, a large proportion of rod-shaped _iACC_MTB forming 2 granules of iACC was systematically observed as previously [25] (Fig. 1B and D). However, a longer magnetic concentration (i.e. up to 4 h) revealed the coexistence of additional _iACC_MTB-like populations, with very different morphologies, sizes, magnetic behaviors, and swimming patterns (Video S1 and Fig. S1). Instead of aggregating at the edge of the drop like most MTB, these bacteria were constantly moving: some had a “ping-pong-like motion” [74], whereas others moved vertically along the drop. Indeed, they aligned along the magnetic field and slowly swam back and forth at the bottom of the drop without reaching the edge of the drop. They have been regularly observed since 2015 in shallow sediments regardless of the season. Their total concentration was estimated to 3 × 10^3^ cells/ml of sediment on average, up to ~10^5^ cells/ml in some samples (Fig. S1D). Most of these undescribed MTB carried additional small refringent inclusions that appeared darkish. They were well distinguishable from the large iACC-like inclusions that are yellowish when observed under the phase contrast of our light microscope. It was hypothesized that these inclusions were sulfur globules which was confirmed later with XEDS analyses (Fig. S2).

To compare the diversity of _iACC_MTB of the sediments with that of the water column, we led a field campaign during October 2021 and carried out a profiling of the MTB populations around the chemocline (Supplementary Notes: Method S1). As it was reported previously [20, 25], an homogenous population of _iACC_MTB (i.e. 2 × 10^3^ cells/ml) was also observed 55 m deep in the water column and further characterized in this study.

### MTB populations forming refractive inclusions are represented by different morphotypes

Given that refringence is a key characteristic used to distinguish _iACC_MTB from other bacteria in shallow sediments, we hypothesized that all yellowish granules observed in all magnetotactic populations were composed of mineral phases. MTB bearing refractive inclusions were classified based on their morphology (i.e. short rods, long rods, ovoid rods), magnetic behavior and swimming patterns (i.e. fast or slow undulatory, helicoidal) and the number of refractive inclusions observed under the optical microscope. When cells of varying sizes shared the same morphology, magnetic behavior and number of inclusions, size was also included as a criterion for classification. A single-cell sorting approach was then applied to characterize further the different morphotypes of these _iACC_MTB-like (i.e. MTB bearing yellowish inclusions resembling those previously described previously [25]). This characterization involved not only identifying the chemical composition of their granules but also investigating and comparing their taxonomy and physiology. More than a dozen of morphotypes were observed in the shallow sediments (Fig. S1C). Only four of them (Fig. 1B and C), representing more than 90% of the _iACC_MTB-*like* in the sediments, were systematically observed in every sampling, at every season and at every site, but with varying relative frequencies (Fig. 1D).

The first _iACC_MTB population described in the sediments previously [25], was classified into the morphotype 1 in this study. Morphotype 1 is a rod-shaped bacterium, measuring 4.2 ± 0.5 μm in length and 1.8 ± 0.3 μm in width, and producing 2 or 4 large refractive inclusions. Morphotype 2 is characterized by longer cells that could reach up to 17 μm in length (12.2 ± 1.9 μm in length and 2.4 ± 0.3 μm in width on average), and by a very fast undulatory swimming pattern with the particularity of performing back and forth movements at the edge of the hanging drop (i.e. “ping pong motion” [74]). Although most of the cells bear five aligned inclusions, up to 12 inclusions were observed in rare cases (Fig. S3). A smaller inclusion was systematically observed at one of the cell poles for cells with more than five inclusions which could be indicative of different growth stages before cell division. The morphology of morphotype 3 is similar to that of some non-magnetotatic iACC-bearing *Achromatium* species described before [26, 32, 75]: cells measure 10.8 ± 1.1 μm in length on average, and are much wider (i.e. 4.4 ± 0.5 μm) than other _iACC_MTB. This bulky morphology probably explained their slow helical motion and their accumulation at the bottom of the hanging drop rather than at its upper edge. They bear up to 10 large, irregular, and nested inclusions that are not as spherical as those in other morphotypes. Morphotype 4 is represented by fast and much smaller cells (i.e. 5.0 ± 0.9 μm long / 2.0 ± 0.3 μm wide) bearing between 10 and 18 unaligned and spherical inclusions. Morphotypes 2, 3 and 4 are thus well distinct from morphotype 1. Although regularly observed, the three new morphotypes represented less than 5% of the total populations in the sediments. The _iACC_MTB population described in the water column previously [20, 25] were classified as the morphotype 5 in this study because they were overall slightly smaller (Fig. 1C) and present others discriminant features compared to morphotype 1 (see below).

### Refractive inclusions in all _iACC_MTB-*like* morphotypes occupy most of the cytoplasmic space and are Ca-rich

**Figure 2:**
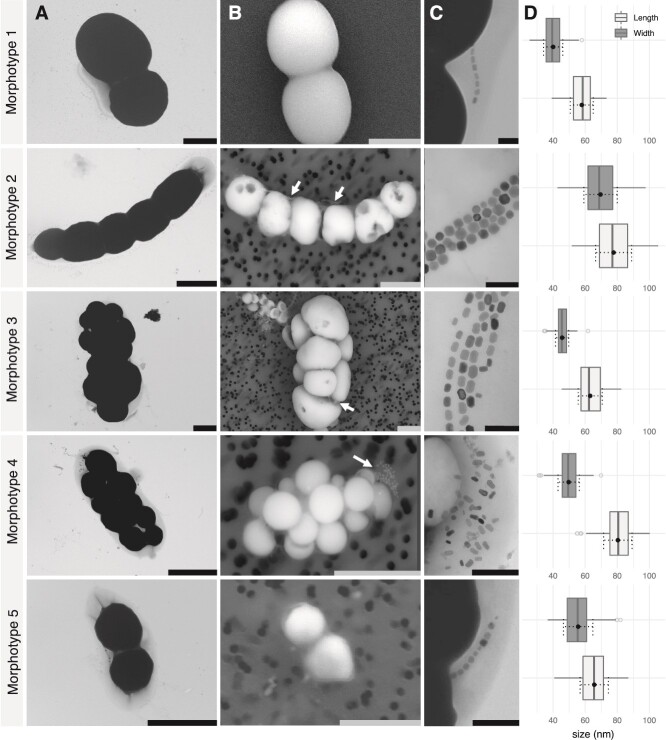
**Ultrastructural characteristics of the main**  _**iACC**_**MTB-like morphotypes with inclusions occupying most of their cell volume.** Representative TEM (**A**) and SEM (**B**) images showing inclusions with a different contrast to the electron beam (black and white respectively) from which iACC diameter was estimated in Fig. 1C. Scales bars represent 2 μm. White arrows indicate the magnetosomes when visible in the SEM images. (**C**) TEM images of corresponding magnetosome chains for each morphotype. Scales bars represent 0.2 μm. Combination of SEM and TEM images highlighted the presence of a single polar flagellum for the morphotype 1 and 4, whereas one and two bundles of polar flagella were observed the morphotypes 3 and 2 respectively (Fig. S7). (**D**) Analysis of magnetosome size distribution for each morphotype. A total of 242, 353, 212, 165 and 210 magnetosomes were analyzed for morphotypes 1, 2, 3, 4 and 5 respectively. Averages and standard deviations are plotted next to boxplots (dotted black lines). The magnetosome shape factor (i.e. ratio length/width) averages were 0.69 ± 0.08, 0.90 ± 0.06, 0.73 ± 0.09, 0.62 ± 0.08 and 0.86 ± 0. 07 (±1SD), respectively. Additional SEM images are given in Fig. S4.

Inclusion size is highly variable between morphotypes, i.e. from 0.8 to 3.9 μm on average, and up to 6.2 μm in length for morphotype 3. However, the size of the inclusions is relatively constant within each morphotype (Fig. 1C). All inclusions are electron dense (Fig. 2) and appear to occupy most of the cytoplasmic space. To more accurately determine the occupancy of iACC, cryo soft X-ray tomography (cryo-SXT) experiments were conducted on ice-vitrified _iACC_MTB. This approach preserves the bacterium’s hydrated state and, through volume segmentation, enables 3D investigation of iACC inclusions volume. They were found to occupy 44–68% of the cytoplasmic space for morphotype 1 (n = 3) and 47–56% for morphotype 2 (n = 3) (Fig. S5). Morphotypes 3 and 4 were also imaged with cryo-SXT. However, due to the strong absorption of the larger iACC inclusions, tomographic reconstruction artifacts hinder accurate volume segmentation and granule volume measurements.

**Figure 3:**
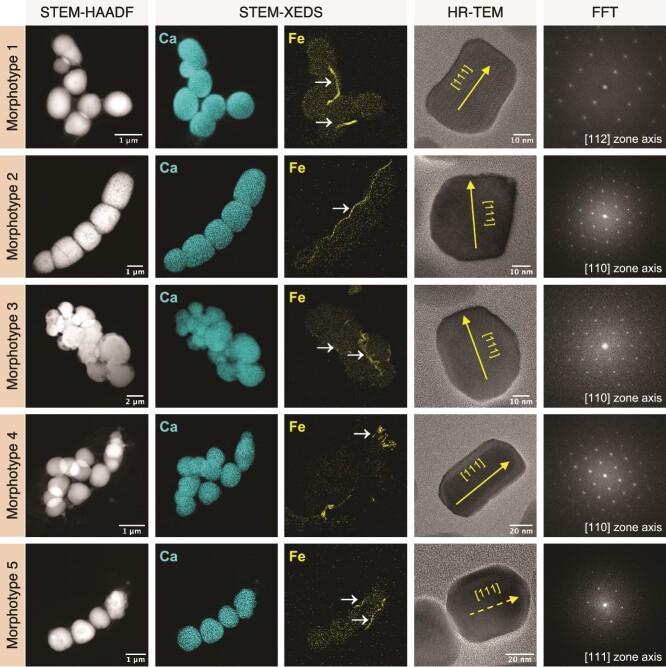
**XEDS elemental mapping of calcium (Ca-K), and iron (Fe-K) from STEM-HAADF images of**  _**iACC**_**MTB representing dominant morphotypes, along with diffraction analysis (HRTEM) of their corresponding magnetosomes.** STEM-XEDS elemental mapping shown are non-background subtracted. The Fe elemental maps display chains of magnetosomes from different morphotypes, indicated by the white arrows. HRTEM images of individual magnetosomes biomineralized by the different _iACC_MTB along with their corresponding fast Fourier transform (FFT) patterns have been indexed based on the magnetite structure. For each HRTEM image, the [111] direction is displayed. For morphotype 1–4, the [111] is in figure plane. For morphotype 5, the [111] direction is out of plane. In a previous study, such analyses for the morphotype 1 have shown that magnetosomes were composed of magnetite (Fe_3_O_4_) nanocrystals and that inclusions were predominantly composed of calcium (Ca) [25]. Here, the same analyzes led to the same conclusions for the other four morphotypes of the sediments and the water column.

In morphotype 1 cells, thin section observations had previously evidenced a lipid bilayer surrounding inclusions [25]. The absence of opening between the inclusions’ lumen and the periplasm space on numerous images suggested that the inclusions were cytoplasmic. Here, we made the same observation for the morphotype 3 (Fig. S6). The inclusions of morphotypes 1 and 3 can thus be considered as cytoplasmic compared to what has been previously reported for *Achromatium* in which iACC inclusions are considered as periplasmic [76]. Scanning transmission electron microscopy (STEM) images of the refractive inclusions in the HAADF mode provided a chemical contrast. They were combined with the determination of elemental composition and distribution by XEDS provided the elemental composition. The relative intensity of the Ca peak compared to that for other elements on spectra obtained for the cells of morphotypes 2 to 5 are comparable with that obtained for morphotype 1 [25]. This indicates that inclusions contained predominantly calcium across all morphotypes (Fig. 3). The two other major peaks observed were indexed as carbon (-C) and oxygen (-O). It was more difficult to get spectra for the small darkish inclusions observed under the optical microscope in most of the _iACC_MTB because they generally dissolved during the dehydration of the cells on the TEM grid. Some of them were still preserved and analyzed in some cells of the morphotype 4 specifically, confirming they were sulfur-rich inclusions (Fig. S2).

HRTEM and elemental analyses confirmed that all morphotypes produce magnetite-based magnetosomes arranged in different ways. Morphotype 1 synthesizes a single chain of 10 to 20 magnetosomes generally organized in the center of the cell [25], whereas morphotype 2 forms a “honeycomb”-like bundle of 3 to 4 chains running along the length of the cell and grouping 160 to 350 magnetosomes. For the third morphotype, cells are characterized by the formation of very large numbers of magnetosomes (i.e. up to 550 magnetosomes per cell), aligned in numerous chains that are generally organized parallel to the longest axis of the cell. In this morphotype, the large Ca-rich inclusions seem to distort locally the magnetosome chains that are close to the cell’s inner membrane. In contrast, morphotype 4 cells synthesize between 10 and 30 disordered magnetosomes aggregated at the cell posterior pole near the flagellum (Fig. S7D). These analyses further refine _iACC_MTB classification by revealing that magnetosomes chains and crystal morphologies were different between populations of the water column and the sediments (i.e. octahedral in morphotype 5 and prismatic in all others, respectively) (Fig. 2 and 3).

### Each morphotype is associated with a genomic group affiliated with divergent families within the *Pseudomonadota*

**Figure 4:**
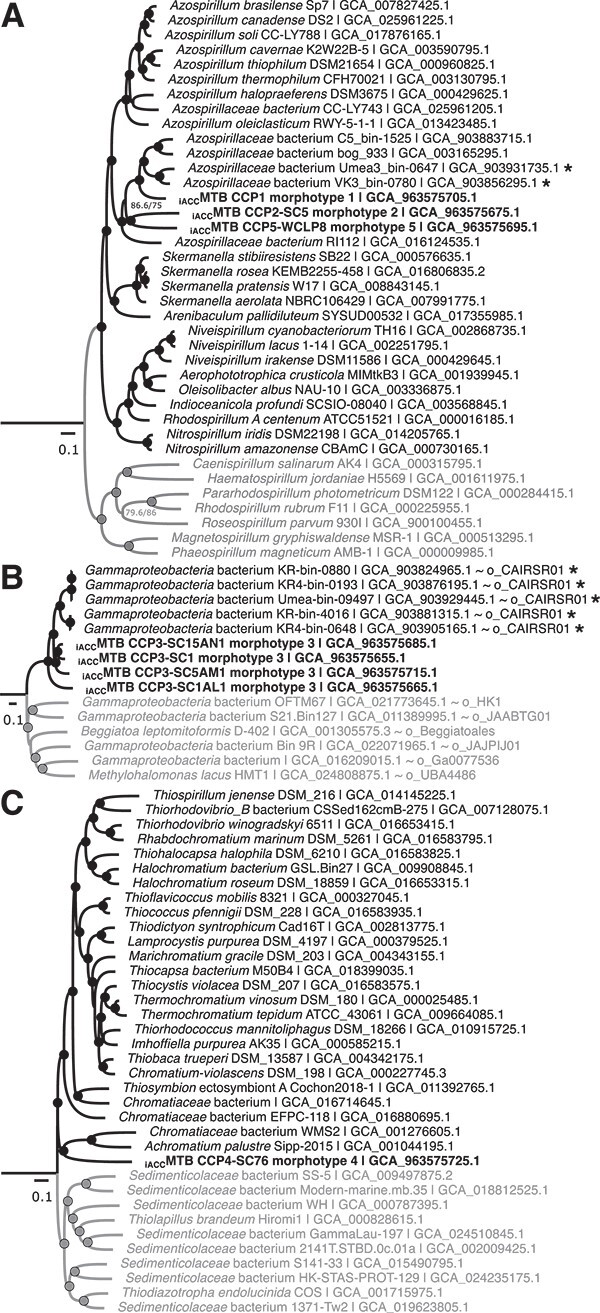
**Maximum-likelihood trees showing the genetic relationships of the**  _**iACC**_**MTB with their closest relatives in the *Pseudomonadota* phylum based on conserved 120 conserved proteins used by the GTDB taxonomy.** (**A**) Phylogenetic tree of the *Azospirillaceae* family (*Alphaproteobacteria*). All genomes of good quality (i.e. > 90% complete with < 5% redundancy according to CheckM v1.0.18 [56] were used, except for the genus *Azospirillum*, for which a taxonomic reduction was done to select the most representative genomes only. The tree was rooted with representative members of several *Rhodospirillaceae* members (grey group), which was the closest monophyletic group of non-symbiotic organisms based on Muñoz-Gomez et al. [92]. Phylogenetic trees shown in (**B**) and (**C**) represent the *Candidatus* order / family CAIRSR01 and the *Chromatiaceae* family (order *Chromatiales*) of the *Gammaproteobacteria* class, respectively. The external group (i.e. the closest monophyletic group) used to root trees (**B**) and (**C**) were identified using a *Gammaproteobacteria* phylogenetic tree built in this study and given in the Fig. S10. The dataset includes all the genomes identified as being in the same group as one or more _iACC_MTB based on GTDB-tk [93] analysis and a selection of genomes of the closest monophyletic group (i.e. *Sedimenticolaceae* for the *Chromatiaceae* tree, and a monophyletic group representing several *Gammaproteobacteria* orders for the CAIRSR01 tree). Branch lengths represent the number of substitutions per site. The circles plotted on the internal nodes represent the statistical support, considered satisfactory when the likelihood rate (aLRT) was greater than 0.95 (estimated from 1000 replicas) and the non-parametric bootstrap value was greater than 80% (estimated from 500 replicas). The _iACC_MTB genomes are shown in bold, whereas the MAGs in which a MGC was assembled are annotated with a “*”. The corresponding Genbank accession numbers are given in the sequence names, along with the corresponding order name “o_” and family name “f_” in GTDB [94] (https://gtdb.ecogenomic.org).

The taxonomic diversity within morphotypes was investigated by amplifying the 16S rRNA gene and by sequencing genomes using several sorted cells (Table S1). For morphotype 5 specifically, which represents _iACC_MTB populations in the water column, a MAG designated as CCP5-WCLP8 was obtained. This MAG contains a 16S rRNA gene sequence and a taxonomic assignment identical to those of cells sorted via micromanipulation. The 16S rRNA gene sequences obtained from the different _iACC_MTB under study were later validated by hybridizing oligonucleotide probes specific to each group of 16S rRNA sequences with cells of each morphotype using FISH and confocal laser scanning microscopy (Supplementary Notes: Method S3 and Fig. S8). Following this procedure, we showed that _iACC_MTB affiliated with morphotypes 2 and 5 cluster with those from morphotype 1 within the *Azospirilllaceae* family (former *Rhodospirillaceae*) (Fig. 4A). Moreover, they also formed a monophyletic group with MAGs from the anoxic zone of the water column of chemically stratified lakes and ponds located at Valkea Kotinen in Finland (VK3_bin-780), Björntjärnen East in Sweden (Umea3_bin-0647), Kuujjuarapik- Whapmagoostui in Canada (C5_bin-1525), and the active permafrost layer at Stordalen Mire in Sweden (BOG_933) [77]. Given the average amino acid identity values (62 and 65% with the closest genomes, Fig. S9) and GTDB-tk analysis, morphotypes 1, 2, and 5 _iACC_MTB likely represent three undescribed genera, which together with candidate genera RI-112, CAIZDL01 and BOG_933, form a sister clade to the *Azospirillum* genus.

**Figure 5:**
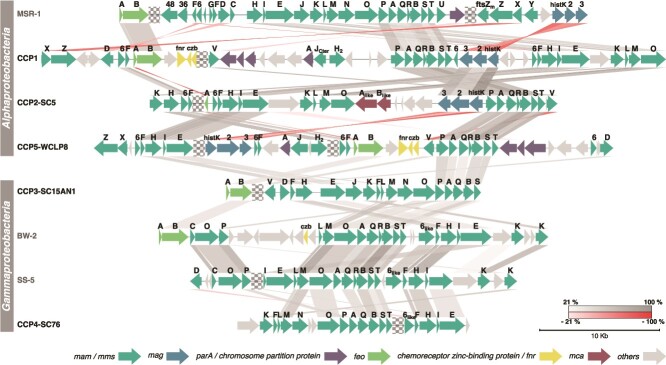
**Conservation of MGC synteny in representative**  _**iACC**_**MTB and the magnetotactic *Pseudomonadota* model strains MSR-1, BW-2, and SS-5.** Genomes are organized by class (*Alphaproteobacteria* and *Gammaproteobacteria*). Names in bold represent the _iACC_MTB genomes sequenced in this study: CCP-1 is the name given to the _iACC_MTB morphotype 1 genome; CCP2-SC-5 to morphotype 2; CCP5-WCLP8 to water column morphotype 5; CCP3-SC15AN1 to morphotype 3; and CCP4-SC76 to morphotype 4. Each arrow represents a gene of a color corresponding to a specific operon in MSR-1 [10]. Grey genes are genes of unknown function or not conserved in MTB. Checkerboards represent mainly truncations and sometimes regions spacing two operons. Sequence identities between reciprocal best hits were estimated with MMseqs2 [95] and are represented by bands, with their intensity reflecting the percentage of identity. Some homologues are not linked due to high sequence divergence and/or the presence of multiple paralogs. Homologues families were then determined by the presence of conserved domains using the microscope platform [73]. A full version of this figure is given in Fig. S13.

Morphotypes 3 and 4 are affiliated with two different orders within the *Gammaproteobacteria* based on the whole genome tree and the GTDB-tk analysis (Fig. S10): a new order named CAIRSR01 proposed by the GTDB taxonomy without official name in nomenclature (i.e. listed in LPSN; https://lpsn.dsmz.de) and the *Chromatiales* order, respectively. Although the size, the morphology and the ultrastructure of morphotype 3 is close to those of non-magnetotactic *Achromatium* species, both groups of _iACC_B belong to very divergent taxa (Fig. S10 and S11). Morphotype 3 is represented by several divergent species, at the genus boundary for some of them, as their genomes share between 68 and 75% of AAI (Fig. S11). Together, they form a monophyletic group clustering with other MAGs that were also recovered from the anoxic zone of the water column of chemically stratified lakes and ponds at Keskinen Rajajärvien and Keskinen Rajajärvi in Finland (KR-bin-0880 / KR-bin-4016 and KR4-bin-0648 / KR4-bin-0193, respectively), and at Björntjärnen East in Sweden (Umea-bin-09497) [77] (Fig. 4B). A phylogenetic tree based on the 16S rRNA gene showed that the morphotype 3 bacteria represented by the CCP3-2020_07_09 sequence belong to the same species as the magnetotactic bacterium GRS-1, which also forms calcium-rich inclusions [21] (Fig. S12B). A full magnetosome gene cluster (MGC) was found in all _iACC_MTB genomes (Fig. 5) and a MGC was also found in several MAGs recovered from other northern stratified lakes that clustered with _iACC_MTB genomes in both *Alphaproteobacteria* and *Gammaproteobacteria* classes (Fig. S13).

Although morphologically very different, morphotype 4 and non-magnetotactic *Achromatium* species, are genetically closer to each other’s and are all affiliated with the *Chromatiaceae* family of the *Chromatiales* order (according to the GTDB classification) (Fig. 4C, Figs. S10 and S11). Based on genome similarities only, the CCP4-SC76 genome represents an undescribed genus as it shares less than 59 AAI% with the closest related genomes, including the _iACC_B *Achromatium palustre* Sipp_2015, *Achromatium* sp. WMS2 and other *A. oxaliferum* related species (Fig. S11B). A tree based on the 16S rRNA gene sequences of *Chromatiaceae* (Fig. S12C) shows that the non-magnetotactic *Achromatium*-like cells observed in the Lake Pavin sediments [26], cluster with freshwater and marine *Achromatium* related species and are also genetically distant from the _iACC_MTB morphotype 4 (Fig. S12C). In these analyses, each genetic group of _iACC_B in the *Chromatiaceae* family seems to be specifically associated to a niche either marine or freshwater.

### Metabolic network modeling predicts different pathways of carbon assimilation in *Alphaproteobacteria* and *Gammaproteobacteria*

**Figure 6:**
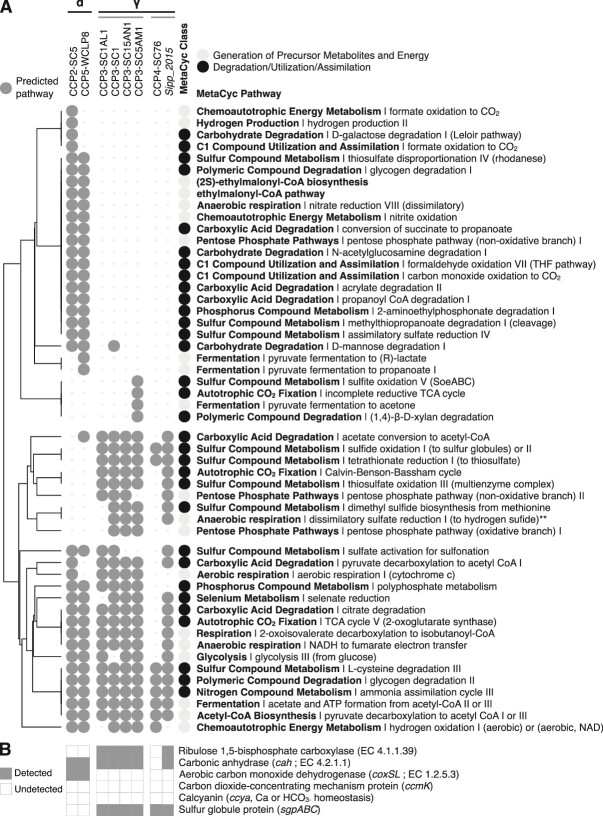
**Heatmap representing selected metabolic pathways and functions involved in energy metabolism, assimilation, utilization or degradation of organic and inorganic compounds, as well as in calcium or sulfur cycling in**  _**iACC**_**MTB.** (**A**) Comparative analysis of MetaCyc metabolic pathways predicted with the pathway tools software in at least one of the _iACC_MTB genomes. The full analysis is given in the Table S2. One of the most complete draft genomes of an _iACC_B affiliated with the *Pseudomonadota* (*Achromatium palustre* Sipp_2015) [75] was also added to the comparison. Genomes are grouped by *Pseudomonadota* classes (*Alphaproteobacteria* and *Gammaproteobacteria*) and orders (CAIRSR01 and *Chromatiales*). Metabolic pathways are organized based on a hierarchical clustering analysis (Euclidean distance clustering algorithm) according to their pair-wise distance. Absence of prediction can be linked to the absence of a single reaction / enzyme / gene mandatory for the pathway prediction. Yet, this absence can be a false negative and be linked to the quality of the draft genome assembly. No draft genome of satisfactory completeness rate was obtained for the species represented by morphotype 1. Note that: (i) the CCP4-SC76 genome is only 60% complete, which explains that less pathways were predicted and (ii), although the dissimilatory sulfate reduction pathway is predicted, it likely realizes the reverse sulfide oxidation in *Gammaproteobacteria*. (**B**) Presence / absence of some marker genes of interest for this study in _iACC_MTB genomes.

Functional annotation of draft genomes and metabolic pathways reconstruction using MetaCyc [70] enabled to draw two very different profiles of carbon incorporation and utilization within *Pseudomonadota* forming iACC (Fig. 6 and Table S2). All can use organic compounds as carbon sources and electron donors, respire oxygen and oxidize hydrogen aerobically. However, they also have a non-canonical form of the TCA cycle that they can use to fix CO_2_ (reverse TCA cycle), in which the 2-oxoglutarate dehydrogenase (EC 1.2.1.105) is replaced by a 2-oxoglutarate synthase (EC 1.2.7.3). Despite these common pathways, _iACC_MTB have class-specific metabolic capacities. The metabolism of _iACC_MTB belonging to the *Gammaproteobacteria* is similar to that previously described for *Achromatium*. Indeed, they likely oxidize reduced sulfur compounds (mainly sulfide and thiosulfate) and generate thiosulfate from the reduction of tetrathionate. Moreover, they can also fix CO_2_ using the Calvin-Benson-Bassham cycle. However, they are unlikely nitrate reducers. This contrasts with the _iACC_MTB belonging to the *Azospirillaceae* that have nitrate reductases (EC 1.7.5.1) and can fix CO_2_ using the rTCA cycle only. Moreover, *Azospirillaceae*  _iACC_MTB also have a nitrite oxidoreductase (NOR) that could provide electrons under oxic conditions or catalyze the reverse reaction under anoxic conditions [78]. *Azospirillaceae*  _iACC_MTB could also produce CO_2_ via methylotrophy and more specifically carboxydotrophy. Indeed, they likely oxidize not only carbon monoxide aerobically, but also formaldehyde, and formate (Fig. 6). Several carbonic anhydrases (EC 4.2.1.1) were annotated, which could catalyze the interconversion of carbon dioxide and bicarbonate. The *ccyA* gene was searched in _iACC_MTB and *Achromatium* genomes as described in [35], but was not detected. No calcium pump (P-type Ca^2+^ transporters) was identified in _iACC_MTB genomes, which does not exclude the existence of some remote homologs. However, as for *Achromatium*, a vacuolar pump (V/A-type H^+^/Na^+^-transporting ATPase; EC:7.1.2.2/7.2.2.1) was found in all the genomes of the CAIRSR01 order, but not in the genomes of *Azospirillaceae*, which harbor F-type H^+^/Na^+^-transporting ATPases only.

## Discussion

Previously, the _iACC_B comprised only a limited number of *Alphaproteobacteria* species, the *Achromatium* genus and several families within the *Cyanobacteriota* phylum (former *Cyanobacteria*). This study, however, has identified several additional species of MTB belonging to distinct genera, families, and orders of *Pseudomonadota,* each exhibiting unique iACC inclusions with previously unobserved sizes, shapes or organizational patterns. This finding further reinforces the polyphyletic distribution of _iACC_MTB within *Bacteria*. Although we observed _iACC_MTB with morphologies closely resembling those of non-magnetotactic *Achromatium*-related species, whole genome analyses did not reveal magnetotactic *Achromatium* species in Lake Pavin. Morphologically and genetically diverse population of _iACC_MTB were often found in the same sample and niche where non-magnetotactic *Achromatium* species had been observed previously [26]. Despite the genomic complexity and the morphological variability observed across all _iACC_MTB and _iACC_B species in *Gammaproteobacteria*, it is striking that they all appear functionally similar, as seen in the *Achromatium* species complex [48, 79, 80]. However, their functional repertoire contrasts totally with that of *Alphaproteobacteria*, which calls into question certain hypotheses on the mechanisms of iACC formation and its potential adaptative roles.

The _iACC_MTB diversity reported here likely represents the tip of the iceberg. Around the same time as the first report of _iACC_MTB [25], a magnetotactic *Rhodospirillaceae* species forming intracellular, poorly crystalline, calcium carbonates was identified in Lake Xingqinggong, Xi’an city, Shaanxi province, China [24]. This suggests that _iACC_MTB are affiliated with several families in *Alphaproteobacteria,* similarly to what we observed in this study for the *Gammaproteobacteria.* Using the criteria applied to classify _iACC_MTB of Lake Pavin, the *Rhodospirillaceae*  _iACC_MTB likely represent a distinct _iACC_MTB morphotype with markedly different ACC inclusions. These bacteria exhibit a spiral or vibrio morphology with an average length of ~2.43 μm and width of ~0.84 μm, and form 2–3 CaCO_3_ inclusions smaller in size (100.4 ± 21.4 nm) than those found in the three *Azospirillaceae* groups of Lake Pavin.

Additional data from other publications suggest that _iACC_MTB are not restricted to Lake Pavin: some *Azospirilllaceae* and *Gammaproteobacteria* form two monophyletic groups with MAGs from the anoxic zone of the water column of chemically stratified lakes and ponds in Finland, Sweden and Canada [77]. The similarity of the environments in which they live and the fact that some of their genomes have a complete or near-complete assembled gene cluster encoding magnetosome formation, strongly suggest that these bacteria are MTB capable of forming iACC. It is also possible that chemically stratified lakes harbor non-magnetotatic bacterial groups forming iACC. Indeed, rare bacteria resembling to _iACC_MTB but swimming randomly (i.e. not attracted by the magnetic fields) were occasionally observed under the light microscope in some magnetic pellets during this study. Additionally, non-magnetotactic *Achromatium* species have been previously observed in Lake Pavin [26]. Phylogenetic trees reconstructed from 16S rRNA gene sequences have highlighted populations close to the _iACC_MTB from Lake Pavin that likely also form iACC. For example, _iACC_MTB of the order CAIRSR01 of *Gammaproteobacteria* was already observed nearly 10 years ago. Taoka *et al*. [21] described a magnetotactic gammaproteobacterium named GRS-1 forming Ca-rich inclusions in a freshwater pond in Kanazawa, Japan. Based on the 16S rRNA gene sequences, the bacterium GRS-1 and the bacterium CCP3-SC1AL1 (morphotype 3) identified in this study belong to the same species, although they differ in the number of iACC inclusions they produce. Although the nature of Ca-rich inclusions in GRS-1 was not investigated at the time, they were likely carbonates. Indeed, GRS-1 bacteria share many other ultrastructural features with morphotype 3 cells: GRS-1 cells are ~13 μm in length and 8 μm in width, form irregularly sized iACC inclusions (2.5–4.5 μm), synthesize over 300 elongated prism-shaped magnetosomes per cell, and swim very slowly following a helical trajectory using a polar flagellum bundle. This observation underscores the decoupling of taxonomy and ultrastructure in non-photosynthetic _iACC_B. Two genetically close species can exhibit markedly different morphologies, such as *Achromatium minus* and *A. oxaliferum* [80]. Conversely, species from distinct genomic orders can share similar if not identical morphologies, as seen with *A. oxaliferum* and the magnetotactic morphotype 3. Therefore, this type of biomineralization appears to be a poor predictor of taxonomy. However, bacteria within the same species tend to produce iACC with the same characteristics in terms of number, shape and organization. This contrasts with magnetosomes in which minerals features can be used to infer genetic proximity [14]. For example, _iACC_MTB morphotypes 2 and 5 share a direct recent common ancestor and have similar magnetosomes chains, but they have very different cell morphologies and iACC features. Although the species-specific nature of magnetosomes supports a well-characterized genetic control [10], this remains to be fully demonstrated for iACC. It is possible that the mineral formation results from passive precipitation of metabolic by-products. In such case, the positioning and partitioning could still be under the control of dedicated homologs of ParA, FtsZ, or MreB-like proteins, similar to those involved in magnetosome biogenesis [10].

Although magnetosomes occupy only a small fraction of the cell’s content, this is not the case for carbonates. In *Achromatium* and in *Azospirillaceae* morphotype 1, the iACC volume has been estimated to account for ~70% and 65% of the total cell volume, respectively [25, 26]. Using the cryo-SXT approach on hydrated cells, which minimizes artifacts, we sought to more accurately estimate this volume for _iACC_MTB. Our measurements indicate that carbonate content falls within the previously measured range but varies between cells of the same morphotype and across morphotypes. With the exception of magnetotactic *Rhodospirillaceae* [24], the iACC inclusions of all *Pseudomonadota* occupy a much larger proportion of cell volume (i.e. ~10 times greater) compared to those in *Cyanobacteriota,* where they account for only about 6–7% of the total cell volume [29].

This substantial storage of intracellular carbon and calcium raises questions about the role(s) of these inclusions in *Pseudomonadota* forming iACC. One of the hypotheses is that they serve as a source of mobilizable inorganic C. The ability to recycle the CO_2_ produced by heterotrophy or methylotrophy, or to concentrate CO_2_ from bicarbonates could represent an adaptation to environments depleted in these substrates. Although this explanation is appealing, it is not entirely satisfactory as previous studies on Lake Pavin showed that organic and inorganic C are not limiting in this habitat [38, 39, 81]. Still, recycling carbon might be advantageous in such conditions if it requires less energy than importing it from the surrounding environment. In addition, the large volume occupied by iACC dramatically impacts the buoyancy of cells by altering cell density. They may function as bacterial statocysts whose movement, perceived by potential mechanoreceptors, could induce a gravitropism. This hypothesis had already been proposed by several authors for intracellular barium sulfates inclusions in several marine eukaryotes [82]. In the specific case of iACC-bearing MTB, we cannot yet exclude the possibility that iACC provide additional assistance in the vertical navigation within the chemical gradients of their environment, complementing the guidance offered by magnetotaxis. Even on such a small scale, optimizing the navigation along vertical gradients is likely crucial for MTB adaptation [83], particularly in such environments where redox gradients can be easily disturbed. Alternatively, it was hypothesized that iACC may serve as a buffering mechanism to counteract proton excess in low pH environments [7, 79]. This hypothesis is supported by observations of small *Achromatium* cells bearing carbonate granules in acidic lakes (pH ~ 5) [80]. However, this possibility seems unlikely in Lake Pavin as the pH is neutral in both sediments and water column where MTB live. Still, iACC could buffer intracellular pH variations resulting from metabolic activities [4, 79]. In *Cyanobacteriota*, the current paradigm is the following: hydrogenocarbonate ions (HCO_3_^−^) are first imported in the cytoplasm and then converted to CO_2_ by carbonic anhydrases. The CO_2_ is then fixed by the enzyme RuBisCO [84]. Then, the conversion of HCO_3_^−^ into CO_2_ releases a hydroxide ion (OH^−^), which reacts with another HCO_3_^−^ to form carbonate ions (CO_3_^2−^). In the presence of sufficient Ca^2+^, this carbonate may precipitate as iACC, thereby balancing the pH increase associated with the conversion of HCO_3_^−^ into CO_2_ [4]. This model also applies to *Achromatium*, as it can fix CO_2_ using the Calvin-Benson-Bassham cycle and two other CO_2_ fixation pathways [79]. In this group of sulfur-oxidizing bacteria*,* it was proposed that iACC could also buffer intracellular pH variations caused by sulfide oxidation to elemental sulfur, a process that consumes protons [7, 75]. On the contrary, oxidation of elemental sulfur into sulfates releases protons which could be buffered by the dissolution of iACC. Our genomic data show that _iACC_MTB taxa in *Gammaproteobacteria* are functionally similar to *Achromatium* supporting the idea that this model might be generalized to _iACC_B in *Gammaproteobacteria*. However, the localization of iACC can call into questions the biological relevance of such a model in some non-photosynthetic _iACC_B [76].

The pH buffering hypothesis is further weakened by *Alphaproteobacteria* characterized in this study as their putative mechanisms for iACC formation differ significantly from those of *Gammaproteobacteria* and *Cyanobacteriota*. Although they can fix CO_2_ using the same variant of the reductive TCA cycle previously described, they are unable to oxidize reduced sulfur compounds and none of their predicted metabolic pathways consume protons; instead, they predominantly release them. This includes aerobic methylotrophy (including carboxydotrophy) and heterotrophy, both of which release CO_2_ and therefore, tend to disfavor ACC precipitation. Similarly, nitrite oxidation, which uses the energy released to support CO_2_ fixation and growth under oxic conditions, does not involve proton consumption [85]. As a result, pH homeostasis cannot be maintained during carbonate formation through these metabolisms alone, unless an additional mechanism is involved. Still, the interconversion of hydrogen and protons via the [NiFe]- and [FeFe]-hydrogenases [86, 87] (EC 1.12.1.2 and EC 1.12.99.6, respectively) could be one of these mechanisms. Indeed, the NADH produced by respiration and glycolysis can be oxidized by protons to form H_2_ and NAD^+^, providing a source of reducing power to support carbon fixation under aerobic conditions in _iACC_MTB. Finally, in this alternative model, environmental conditions such as H_2_ / O_2_ / CO_2_ partial pressures, along with organic carbon and nitrites concentrations, could regulate iACC formation and dissolution in *Alphaproteobacteria*.

Metabolism alone cannot fully explain the bacterium’s ability to produce iACC. Indeed, many of the metabolic pathways predicted in _iACC_MTB genomes, occur in other bacteria that do not accumulate a massive amount of CaCO_3_. Therefore, additional functions and genes might be required. Given that iACC of magnetotactic *Alphaproteobacteria* and *Gammaproteobacteria* are both surrounded by a lipid bilayer, the cell may have evolved a machinery to regulate proton balance and Ca / iC concentrations in the compartment where iACC are formed. This process is likely facilitated by antiporters and transporters that import and / or export chemicals between the environment, the cytoplasm, and the inclusion lumen. _iACC_MTB would need to incorporate massive amounts of Ca, similarly to *Achromatium* whose Ca content can reach an average of 2.6 ng.cell^−1^ [27]. In addition to cation-transporting pumps, some vacuolar-type pumps (V/A-type H^+^/Na^+^-transporting ATPases) were found in most of the _iACC_MTB genomes. In eukaryotes, these pumps are typically associated with organelle membranes [88]. Some *Achromatium* genomes also harbor a type V Ca^2+^-ATPase that may be linked to the import of Ca^2+^ ions from the cytoplasm, across the membrane and towards the lumen of the iACC-containing inclusions [76]. In contrast, no functional equivalent ATPases were found in *Cyanobacteriota* [35], which lack inclusions bounded by lipid bilayers [89]. In *Cyanobacteriota*, a new gene family called *ccyA* was shown to play a role in iACC formation [35]. Despite evidence of its involvement in calcium homeostasis, its specific function remains unclear. This gene has not been found in any of the _iACC_MTB genomes analyzed in this study, nor in *Achromatium* genomes published previously [48, 90]. At this stage, it is difficult to draw conclusions from this observation. It is possible that a non-homologous gene, or a gene with remote homology, performs a similar function in _iACC_MTB. Alternatively, iACC formation could also be driven by mechanisms distinct from those in *Cyanobacteriota*. Given that the genome dataset used in this study is still incomplete and relatively small, it is not yet possible to carry out a comprehensive comparative genomics analysis to identify candidate markers and modular architectures specific of iACC formation as it was done for *Cyanobacteriota* [35].

To conclude, this study contributes to increase the genetic and functional diversity associated with intracellular carbonatogenesis. Unlike to magnetosome formation, the absence of molecular determinants for iACC formation makes unclear whether this biomineralization has one single evolutionary origin and experienced horizontal gene transfers, or if it has emerged multiple times and involves different mechanisms in *Cyanobacteriota* and the two classes of *Pseudomonadota*. Moreover, it is still uncertain whether iACC inclusions have the same function(s) across all taxa or respond to the same environmental pressures. A finely resolved profiling of microbial communities and geochemistry in these ecosystems would enable to identify which conditions specifically favor the proliferation of each _iACC_B species in aquatic sediments. Given the fact that their electron donors and acceptors are likely colocalized over few millimeters to few centimeters, species of *Gammaproteobacteria* and *Alphaproteobacteria* might coexist in the same sediment depths. In the water column, the absence of magnetotactic *Gammaproteobacteria* could be explained by the size of the gradients, as several dozens of centimeters separate their electron donors and acceptors. However, the fact that two different genera of *Alphaproteobacteria* outcompete the others _iACC_MTB in both sediments and the water column, suggests the existence of other environmental factors structuring populations. Based on previous geochemistry analyses [20, 25], calcium concentration is not a structuring factor as levels in Lake Pavin are within a normal range when compared to other freshwater lakes, neither limiting nor excessive. However, in meromictic lakes such as lake Pavin, bacteria can access diverse and abundant sources of CO_2_ and C1 compounds either of mantle origin or produced by heterotrophs, methanotrophs and methylotrophs [39, 81, 91]. According to their genomes, these substrates seem to be particularly important for non-photosynthetic _iACC_B and may explain their abundance in sediments and water column of chemically stratified lakes such as Lake Pavin as well as those from the located in the boreal and subarctic regions [77].

## Supplementary Material

20241219_Supplementary_Information_wrae260

Table_S1_List_sequenced_genomes_wrae260

Table_S2_All_Metabolic_pathways_wrae260

20241219_Supplementary_Notes_wrae260

Data_S1_Gammaproteobacteria_Tree_Newick_format_wrae260

Video_S1_wrae260

## Data Availability

Data generated or analyzed during this study are included in this published article and its Supplementary Information files. The sequencing data were deposited on public databases. The 16S rRNA gene amplicon sequences were deposited in the NCBI Genbank database under the accession numbers OR539775-OR539792, OR761886-OR761890, OR762248-OR762250, and OR770588-OR770590. The genome assemblies were deposited in the NCBI BioProject database under the accession number PRJEB67679.
