# Supplementary material for: Magnetotactic bacteria affiliated with diverse Pseudomonadota families biomineralize intracellular Ca-carbonate
Source: ISME J. 2025 Jan 7;19(1):wrae260. doi: 10.1093/ismejo/wrae260 (PMC11773610; doi:10.1093/ismejo/wrae260)
Supplement: 20241219_Supplementary_Information_wrae260 [file 20241219_supplementary_information_wrae260.pdf]

Supporting information

Magnetotactic bacteria affiliated with diverse *Pseudomonadota* families biomineralize intracellular Ca-carbonate

Camille C. Mangin[1], Karim Benzerara[2], Marine Bergot[1], Nicolas Menguy[2], Béatrice Alonso[1], Stéphanie Fouteau[3], Raphaël Méheust[3], Daniel Chevrier[1], Christian Godon[1], Elsa Turrini[1], Neha Mehta[2], Arnaud Duverger[2], Cynthia Travert[2], Vincent Busigny[4], Elodie Duprat[2], Romain Bolzoni[1,2], Corinne Cruaud[5], Eric Viollier[6], Didier Jézéquel[4,7], David Vallenet[3], Christopher T. Lefèvre[1] & Caroline L. Monteil[1*]

[1] *Université Aix-Marseille, CNRS, CEA, UMR7265 Institut de Biosciences and Biotechnologies d'Aix-Marseille, CEA Cadarache, F-13108 Saint-Paul-lez-Durance, France.* [2] *Sorbonne Université, Institut de Minéralogie, de Physique des Matériaux et de Cosmochimie (IMPMC) - UMR 7590 CNRS MNHN - 4, place Jussieu - BC 115 - 75252 Paris Cedex 5.* [3] *LABGeM, Génomique Métabolique, CEA, Genoscope, Institut François Jacob, CNRS, Université d'Évry, Université Paris-Saclay, Evry, France.* [4] *Université Paris Cité, Institut de Physique du Globe de Paris, CNRS, F-75005, Paris, France.* [5] *Genoscope, Institut de biologie François Jacob, CEA, Université Paris-Saclay, Evry, France.* [6] *LSCE, CEA/CNRS/UVSQ/IPSL, Université Paris Saclay, Université Paris Cité, France 91191 Gif-sur-Yvette Cedex.* [7] *UMR CARRTEL, INRAE-USMB, Thonon, France*

*Corresponding author: Caroline L. Monteil, Université Aix-Marseille, CNRS, CEA, UMR7265 Institut de Biosciences and Biotechnologies d'Aix-Marseille, CEA Cadarache, F-13108 Saint-Paul-lez-Durance, France.
Email: caroline.monteil@cea.fr

Contents

1. **Figures**

Fig. S1. Observation and quantification of MTB populations with large refractive inclusions in the shallow sediments (10 cm to 1 m deep) of Lake Pavin

Fig. S2. XEDS elemental mapping of calcium (Ca-K), sulfur (S-K), oxygen (O-K) and iron (Fe-K) from STEM-HAADF images of the $_{iACC}$MTB morphotype 4

Fig. S3. Optical microscopy images showing the "sub-morphotypic" diversity of morphotype 2 within the *Azospirillaceae*

Fig. S4. Additional SEM images showing the cell organization with electron dense iACC inclusions

Fig. S5. Virtual slices (in z-plane) of cryo-soft X-ray tomography (cryo-SXT) data for $_{iACC}$MTB with examples of volume segmentation for iACC granules (in blue) and cytoplasmic space (in yellow)

Fig. S6. TEM images of thin sections showing the ultrastructure of a $_{iACC}$MTB of morphotype 3 (*Gammaproteobacteria*)

Fig. S7. TEM images showing the position of the flagella (red arrows) of morphotype 1 (A), 2 (B), 3 (C) and 4 (D)

Fig. S8. Fluorescence *in situ* hybridization (FISH) performed for the 3 new $_{iACC}$MTB morphotypes affiliated with the *Gammaproteobacteria* and *Alphaproteobacteria* that were observed in the sediment

Fig. S9. Heatmap of the average Amino Acid Identity (AAI) estimated from pairwise genome comparisons of genomes used in the trees of the Figure 4 (*Azospirillaceae* family of the *Alphaproteobacteria*, CAIRSR01 order and *Chromatiaceae* family of the *Gammaproteobacteria*, respectively

Fig. S10. Maximum-likelihood tree of the *Gammaproteobacteria* class based on 120 conserved bacterial markers used for the GTDB classification showing the distribution of morphotype 3 and 4 in two different orders (*i.e.*, CAIRSR01 and *Chromatiales*, respectively)

Fig. S11. Maximum-likelihood tree of the *Chromatiaceae* family (A) and heatmap of AAI relatedness among the ${}_{iACC}$B genera including *Achromatium* related species (B)

Fig. S12. Phylogenetic trees based on the 16S rRNA gene sequence showing the relationships of ${}_{iACC}$MTB with other environmental bacteria

Fig. S13. Conservation of magnetosome gene cluster (MGC) synteny of the ${}_{iACC}$MTB sequenced in this study, the MAGs with which they cluster in phylogenetic trees and the magnetotactic *Pseudomonadota* model strains MSR-1, BW-2 and SS-5

2. Tables

Table S1. List of ${}_{iACC}$MTB genomes used in this study

Table S2. Comparative analysis of metabolic pathways predicted in at least in one of the ${}_{iACC}$MTB genomes based on the MetaCyc pathways database

3. Videos

Video S1. Light microscope observation of a cell pellet magnetically concentrated from the sediments of Lake Pavin

4. Data

Data S1. Maximum-likelihood tree of the *Gammaproteobacteria* class

5. References

1. Figures

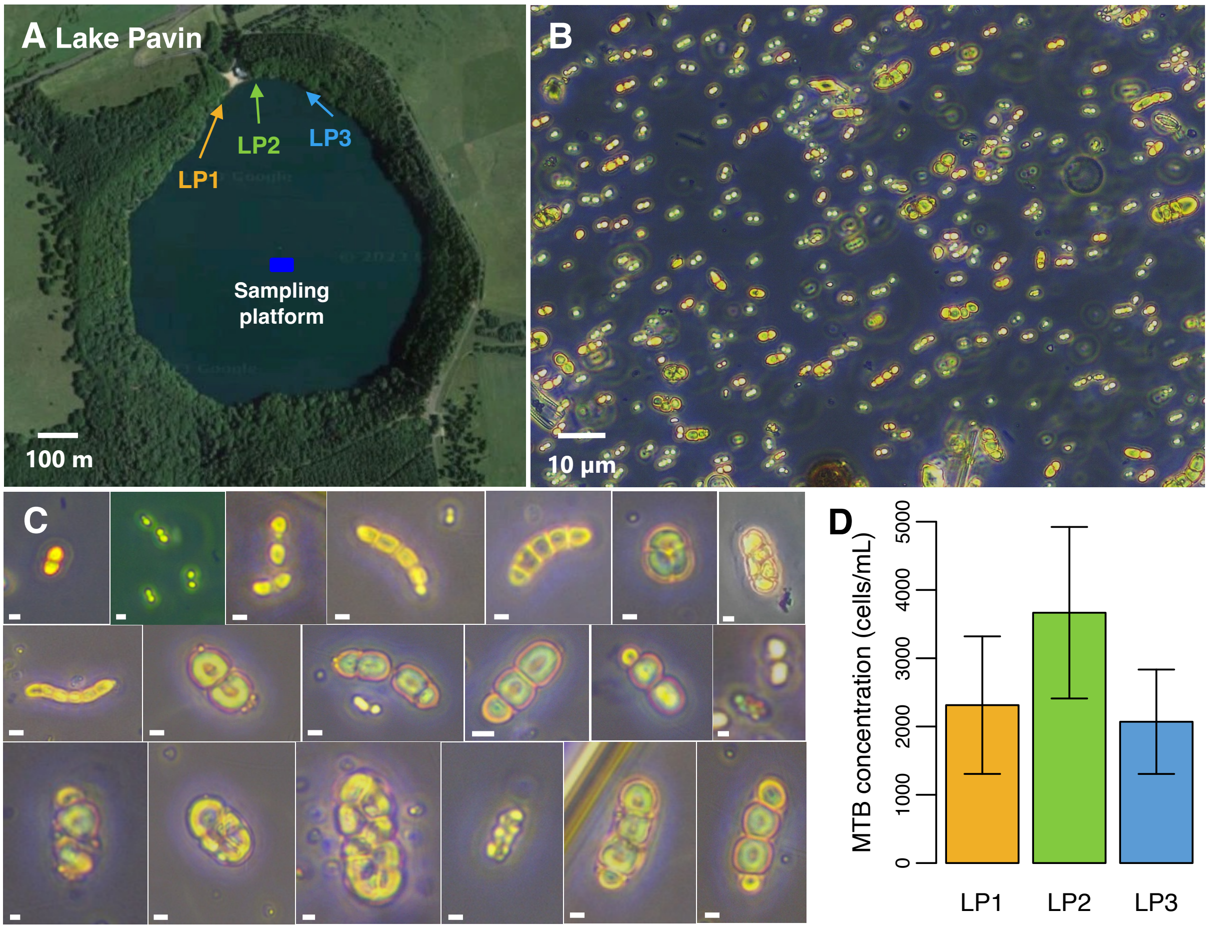

Figure S1. Observation and quantification of MTB populations with large refractive inclusions in the shallow sediments (10 cm to 1 m deep) of Lake Pavin. (**A**) Localization of selected sites sampled from the shore: LP1; 45°29'56.2"N 2°53'10.1"E, LP2; 45°29'58.0"N 2°53'15.6"E, and LP3; 45°29'57.5"N 2°53'18.8"E. Site and sediment characteristics present some differences. Sediments of LP1 (acronym for "Lake Pavin n°1") are shallow, both granular and sandy. Site LP2 is in a totally sheltered environment with deciduous trees, less exposed to light and seems to be covered with decaying material, thus richer in organic matter (e.g. dead leaves, algae, plants), and the sediments are muddier. Site LP3, quite far from the touristic influence, is represented by muddy sediments with lots of organic matter and pieces of submerged wood. (**B**) Representative light microscopy image of north-seeking MTB diversity in sediments, most of them bearing highly refractive granules. (**C**) Light microscopy images representing different MTB morphotypes forming highly refractive inclusions. Scales bars represent 1 μm. (**D**) Concentrations of MTB forming large refractive inclusions in the sediments estimated from the method described in Supplementary information 2 (n = 3 / site). Concentrations were on average 2.7×10^3 cells per ml of sediment up to ~ 10^5 cells per ml in

some samples. If we roughly estimate the pore water to represent 50% of the sandy sediment, then this average concentration could reach up to 5.4×10^3 cells per ml of poral water.

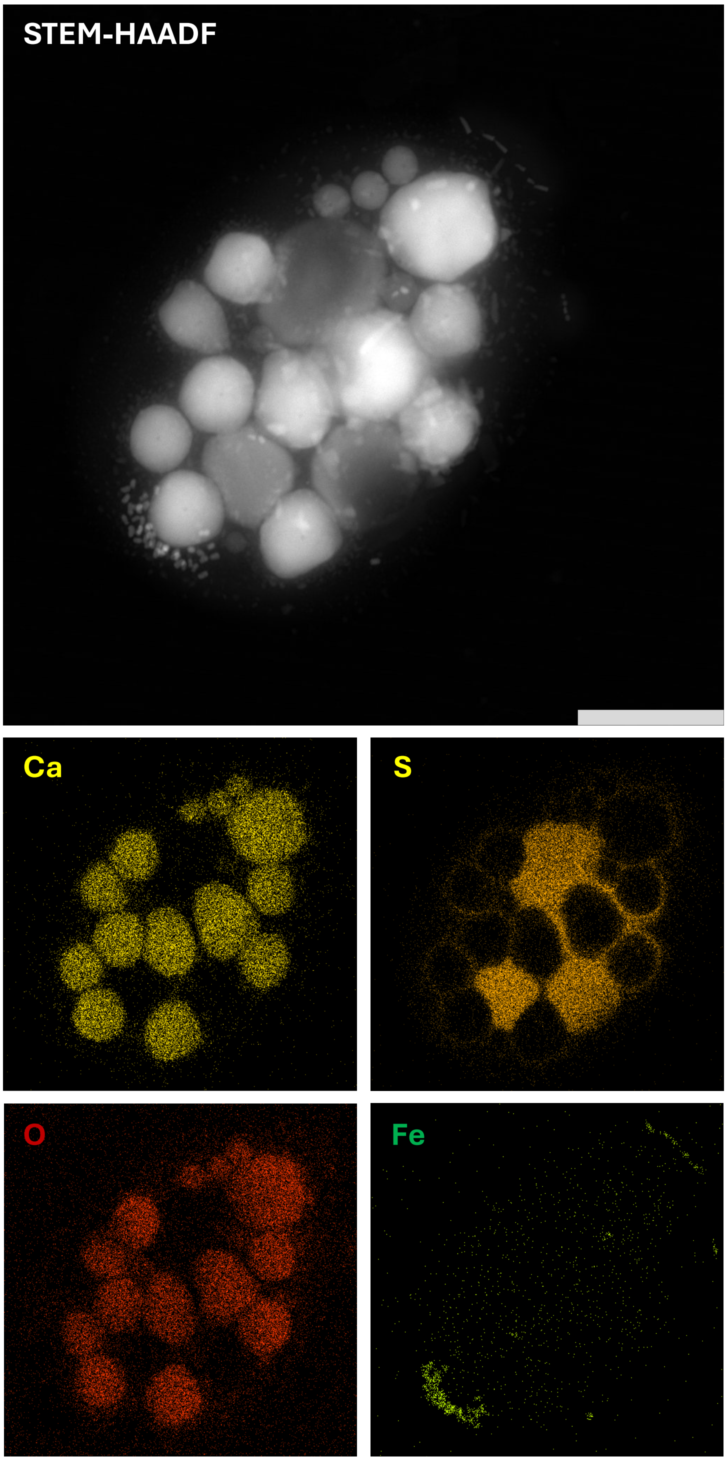

Figure S2. XEDS elemental mapping of calcium (Ca-K), sulfur (S-K), oxygen (O-K) and iron (Fe-K) from STEM-HAADF images of the $_{\text{iACC}}$MTB morphotype 4. This analysis for the morphotype 4 shows three types of inclusions: Fe-rich magnetosomes, Ca-rich iACC and S-rich sulfur inclusions. Scale bar represents 1 μm.

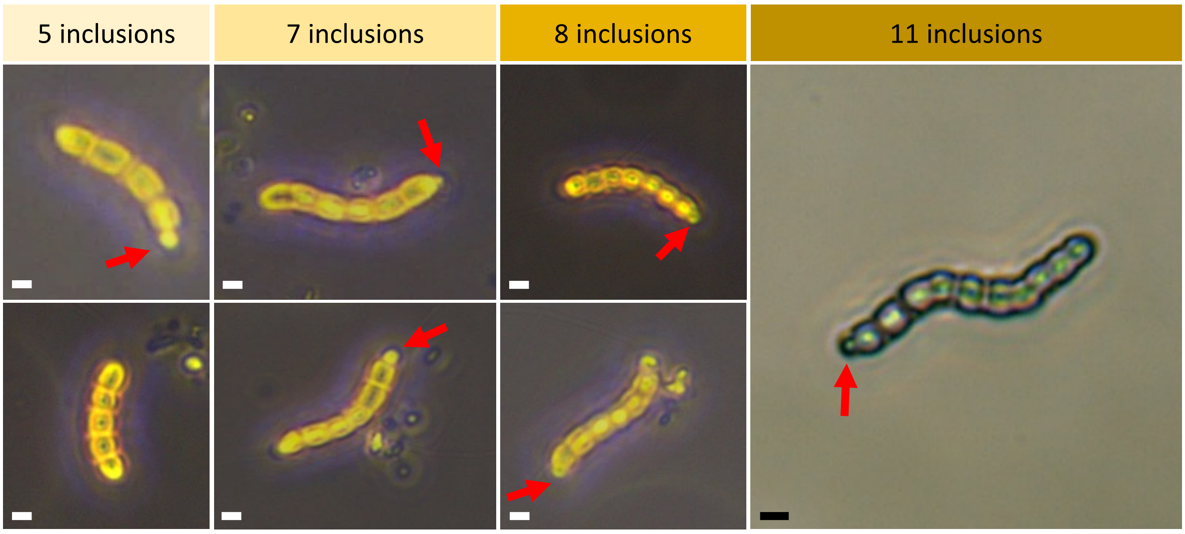

Figure S3. Optical microscopy images showing the "sub-morphotypic" diversity of morphotype 2 within the *Azospirillaceae*. For cells with more than 5 inclusions, a smaller inclusion has been systematically observed at one of the cell tips suggesting that cells with different inclusions might represent different growth stages before cell division when the cell has formed 11 inclusions. If confirmed, this iACC inheritance pattern would be different from that proposed for the morphotype 1, in which two daughter iACC granules are formed at the cell center before cell division. Red arrows indicate small inclusions in the making. Gray scale bars represent 2 µm and the black scale bar represents 3 µm.

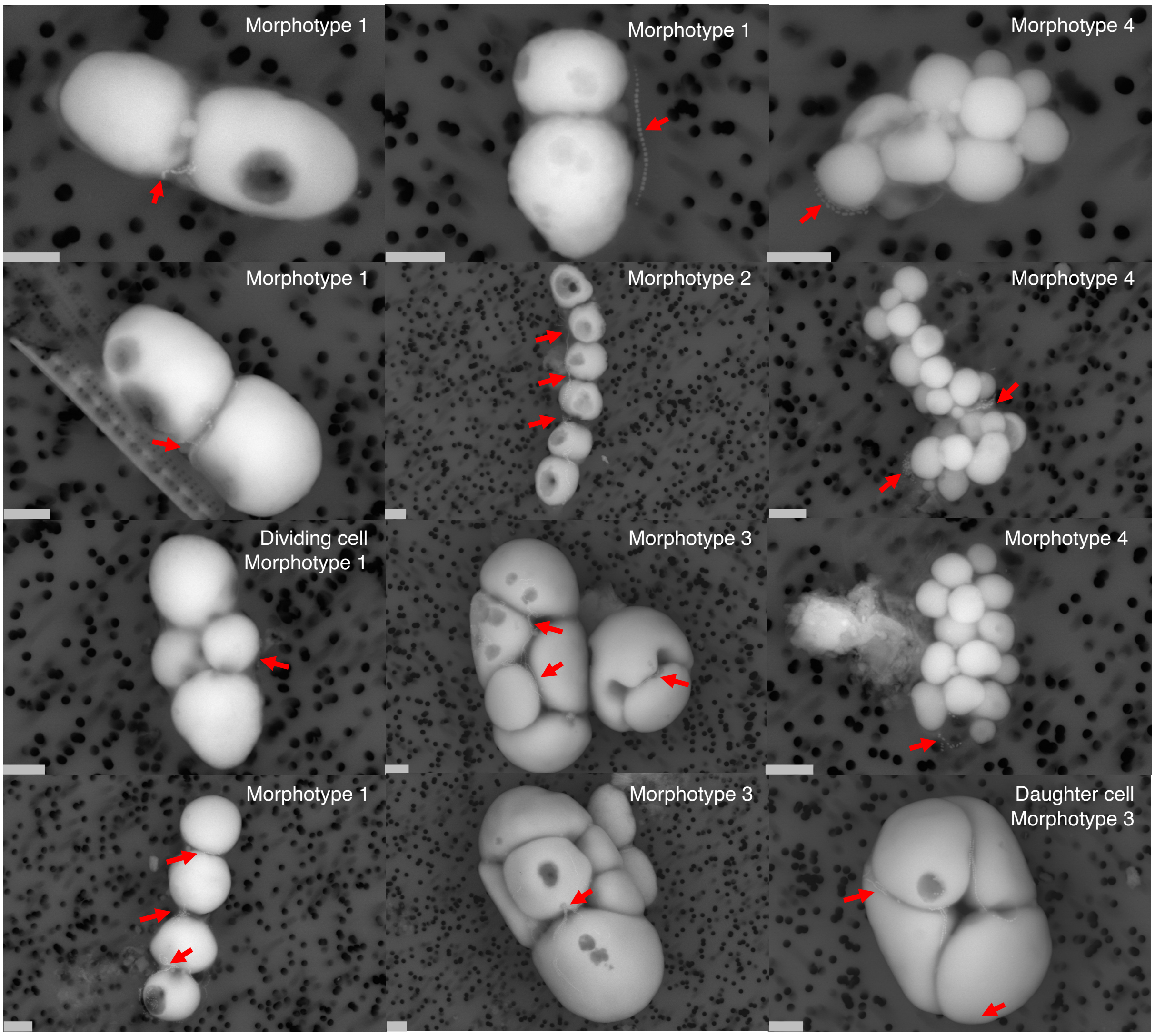

Figure S4. Additional SEM images showing the cell organization with electron dense iACC inclusions. Magnetosomes chains are pointed out by red arrows. Gray scales bars represent 1 μm.

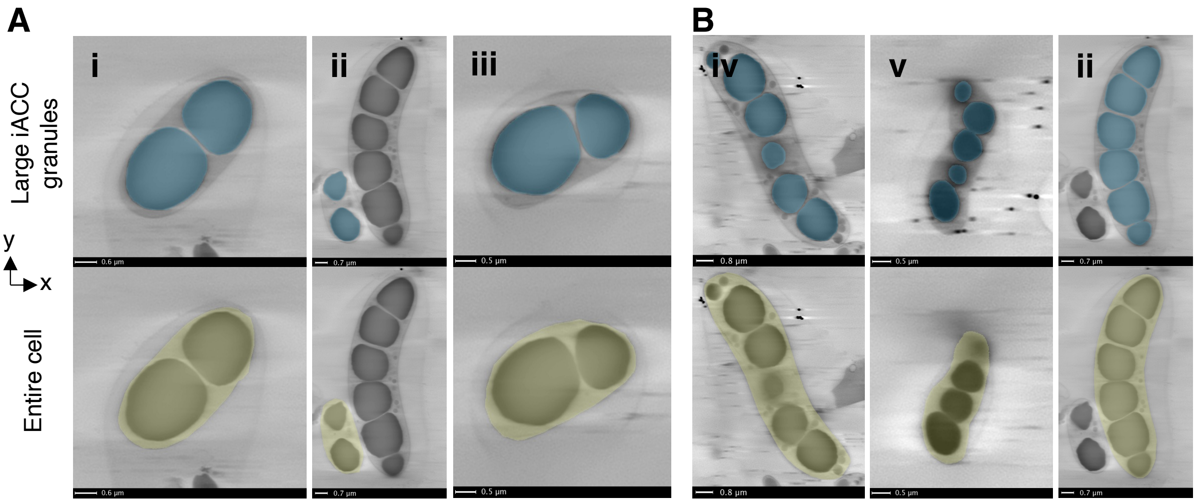

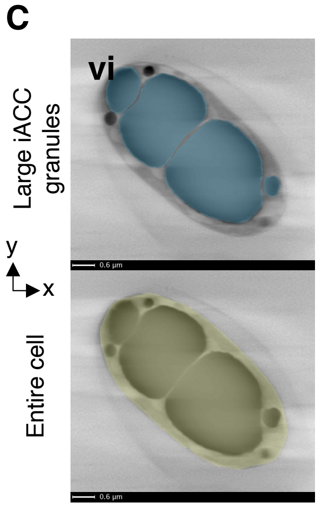

D

Morphotype	Dataset	iACC volume (μm^3)	Cell volume (μm^3)	iACC occupancy
1	(**A**)i	7.4	11.3	66 %
	(**A**)ii	4.3	9.9	44 %
	(**A**)iii	6.5	9.4	68 %
2	(**B**)iv	20.7	44.0	47 %
	(**B**)v	2.3	4.6	50 %
	(**B**)ii	30.4	53.8	56 %
Unknown	(**C**)vi	16.0	24.6	65 %

Figure S5. Virtual slices (in z-plane) of cryo-soft X-ray tomography (cryo-SXT) data for $_{iACC}$MTB with examples of volume segmentation for iACC granules (in blue) and cytoplasmic space (in yellow). Panels (**A**) to (**C**) datasets for the morphotype 1, 2 and an undescribed morphotype respectively (likely a gammaproteobacterium). Dataset numbers are indicated at the top left corner. (**D**) Summary of iACC inclusion and cytoplasmic space volume retrieved from material segmentation of cryo-soft X-ray tomography (cryo-SXT) imaging.

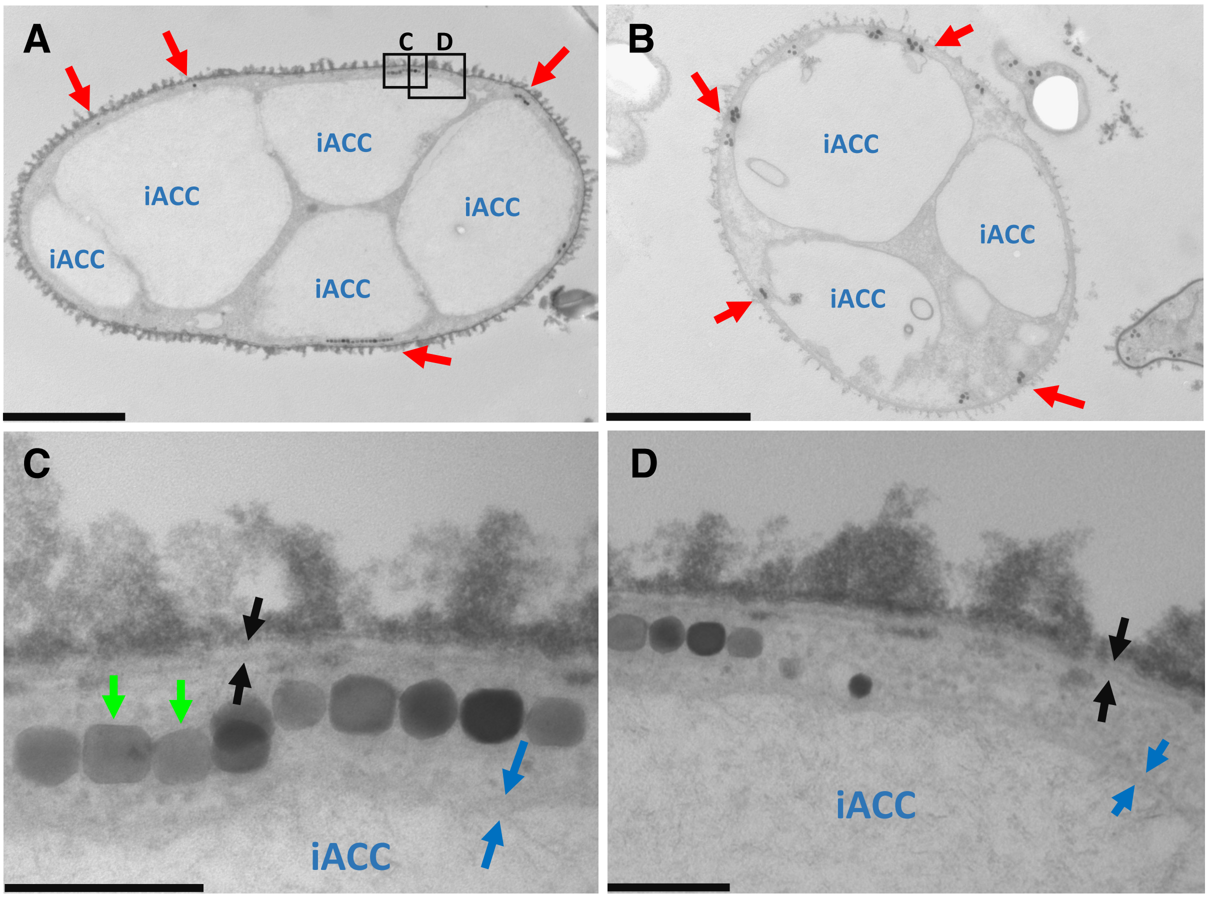

Figure S6. TEM images of thin sections showing the ultrastructure of a $_{iACC}$MTB of morphotype 3 (*Gammaproteobacteria*). (**A**) Longitudinal thin-section image of one cell and (**B**) transverse thin-section image of another cell. The large iACC inclusions, indicated in blue, disaggregated during thin-section preparation. Red arrows show the disorganized magnetosome chains in the vicinity of the periplasm. (**C**) and (**D**) Zoom in on periplasmic zones corresponding to the framed areas in panel A. The presence of a membrane surrounding magnetosomes crystals is indicated in green. The double cell membrane typical of Gram-negative bacteria is shown with black arrows, whereas those in blue point out a double membrane surrounding the iACC inclusions. Scales bars of panels A and B represent 2 μm, scale bars of panels C and D represent 200 nm.

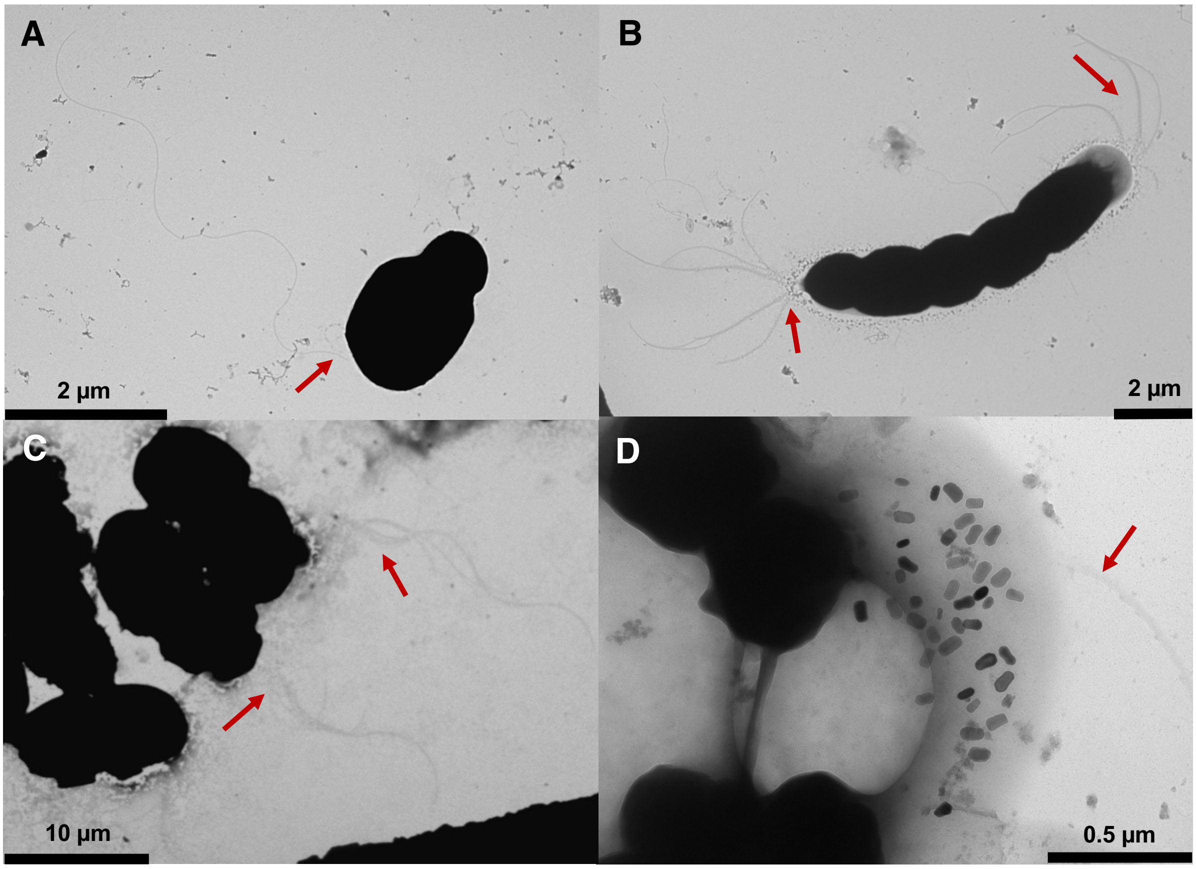

Figure S7. TEM images showing the position of the flagella (red arrows) of morphotype 1 (A), 2 (B), 3 (C) and 4 (D).

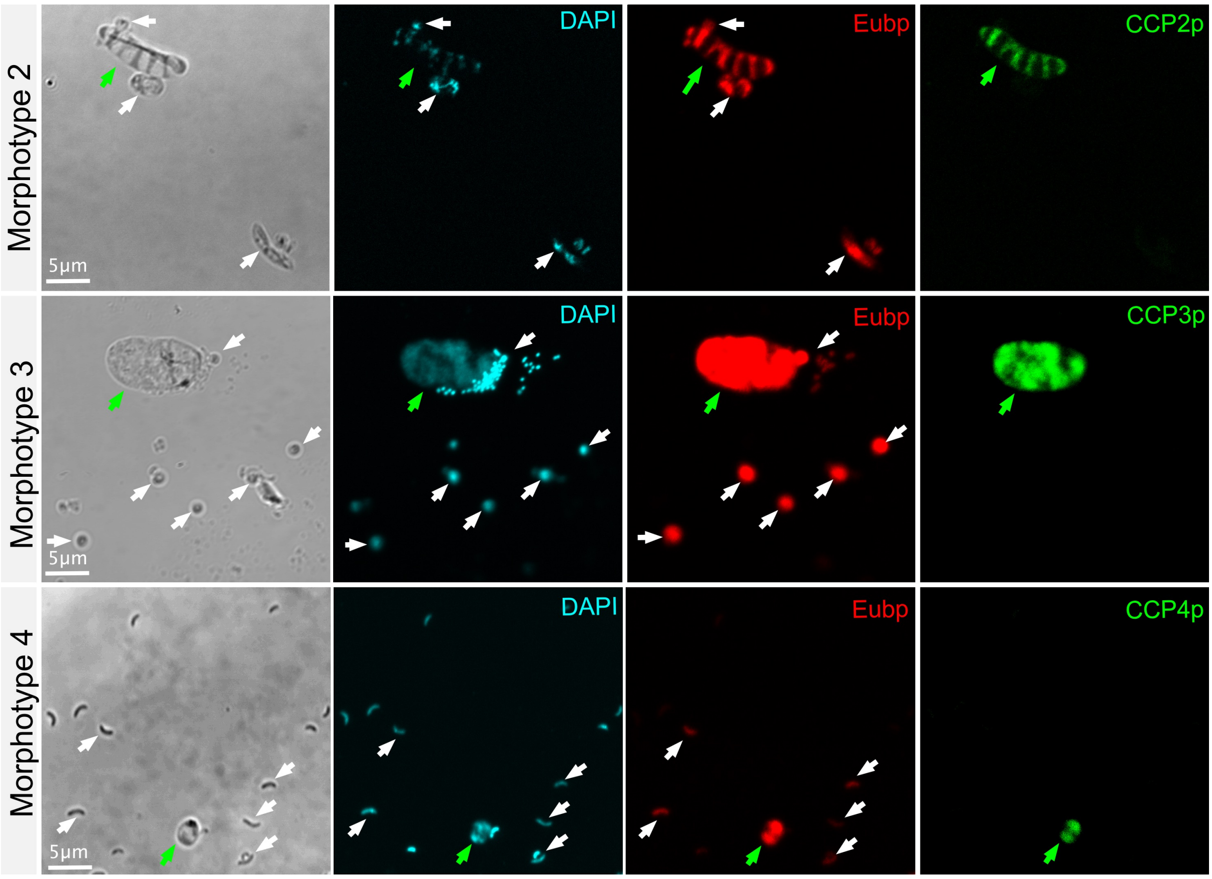

Figure S8. Fluorescence *in situ* hybridization (FISH) performed for the 3 new $_{\text{iACC}}$MTB morphotypes affiliated with the *Gammaproteobacteria* and *Alphaproteobacteria* that were observed in the sediment. For each morphotype, it was possible to obtain transmitted light confocal microscope images (left panel), and laser scan images of cells 1) labeled with 4',6-diamidino-2-phenylindole (DAPI), 2) hybridized with a bacteria-specific probe (Eubp), and finally 3) hybridized with the probe specific to the morphotype of interest (see details in Supplementary Information 3). White arrows show MTB as controls whereas green arrows show $_{\text{iACC}}$MTB. Genomic species were validated for the morphotype1 previously [1]. No FISH experiment was performed for cells of morphotype 5 because species form a monophyletic group with those of morphotype 1 and 2.

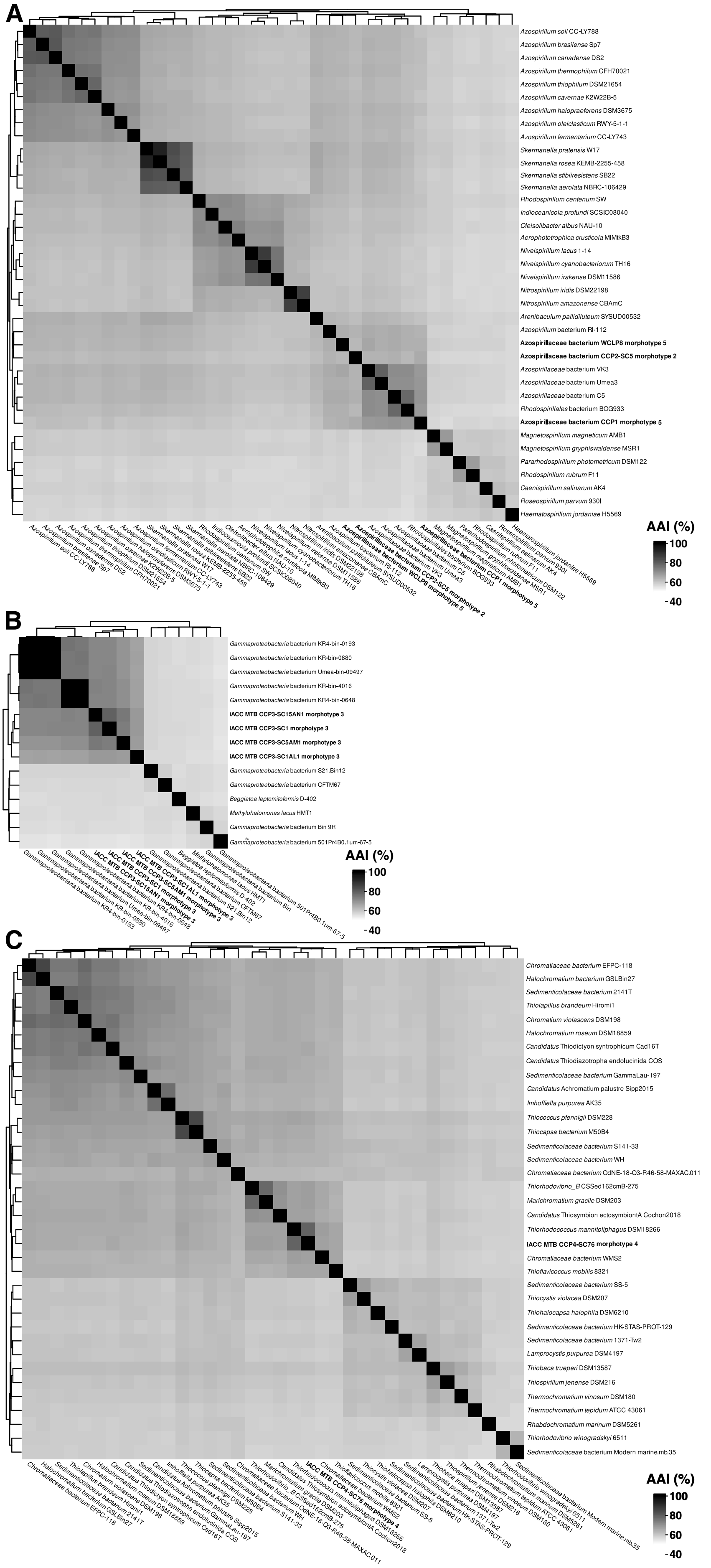

A
Azospirillum soli CC-LY788
Azospirillum brasilense Sp7
Azospirillum canadense DS2
Azospirillum thermophilum CFH70021
Azospirillum thiophilum DSM21654
Azospirillum cavernae K2W22B-5
Azospirillum halopraeferens DSM3675
Azospirillum oleiclasticum RWY-5-1-1
Azospirillum fermentarium CC-LY743
Skermanella pratensis W17
Skermanella rosea KEMB-2255-458
Skermanella stibiiresistens SB22
Skermanella aerolata NBRC-106429
Rhodospirillum centenum SW
Indioceanicola profundi SCSIO08040
Oleisolibacter albus NAU-10
Aerophototrophica crusticola MIMtkB3
Niveispirillum lacus 1-14
Niveispirillum cyanobacteriorum TH16
Niveispirillum irakense DSM11586
Nitrospirillum iridis DSM22198
Nitrospirillum amazonense CBAmC
Arenibaculum pallidiluteum SYSUD00532
Azospirillum bacterium RI-112
Azospirillaceae bacterium WCLP8 morphotype 5
Azospirillaceae bacterium CCP2-SC5 morphotype 2
Azospirillaceae bacterium VK3
Azospirillaceae bacterium Umea3
Azospirillaceae bacterium C5
Rhodospirillales bacterium BOG933
Azospirillaceae bacterium CCP1 morphotype 5
Magnetospirillum magneticum AMB1
Magnetospirillum gryphiswaldense MSR1
Pararhodospirillum photometricum DSM122
Rhodospirillum rubrum F11
Caenispirillum salinarum AK4
Roseospirillum parvum 930I
Haematospirillum jordaniae H5569
AAI (%)
100
80
60
40
B
Gammaproteobacteria bacterium KR4-bin-0193
Gammaproteobacteria bacterium KR-bin-0880
Gammaproteobacteria bacterium Umea-bin-09497
Gammaproteobacteria bacterium KR-bin-4016
Gammaproteobacteria bacterium KR4-bin-0648
iACC MTB CCP3-SC15AN1 morphotype 3
iACC MTB CCP3-SC1 morphotype 3
iACC MTB CCP3-SC5AM1 morphotype 3
iACC MTB CCP3-SC1AL1 morphotype 3
Gammaproteobacteria bacterium S21.Bin12
Gammaproteobacteria bacterium OFTM67
Beggiatoa leptomitoformis D-402
Methylohalomonas lacus HMT1
Gammaproteobacteria bacterium Bin 9R
Gammaproteobacteria bacterium 501Pr4B0.1um-67-5
AAI (%)
100
80
60
40
C
Chromatiaceae bacterium EFPC-118
Halochromatium bacterium GSLBin27
Sedimenticolaceae bacterium 2141T
Thiolapillus brandeum Hiromi1
Chromatium violascens DSM198
Halochromatium roseum DSM18859
Candidatus Thiodictyon syntrophicum Cad16T
Candidatus Thiodiazotropha endolucinida COS
Sedimenticolaceae bacterium GammaLau-197
Candidatus Achromatium palustre Sipp2015
Imhoffiella purpurea AK35
Thiococcus pfennigii DSM228
Thiocapsa bacterium M50B4
Sedimenticolaceae bacterium S141-33
Sedimenticolaceae bacterium WH
Chromatiaceae bacterium OdNE-18-Q3-R46-58-MAXAC.011
Thiorhodovibrio_B CSSed162cmB-275
Marichromatium gracile DSM203
Candidatus Thiosymbion ectosymbiontA Cochon2018
Thiorhodococcus mannitoliphagus DSM18266
iACC MTB CCP4-SC76 morphotype 4
Chromatiaceae bacterium WMS2
Thioflavicoccus mobilis 8321
Sedimenticolaceae bacterium SS-5
Thiocystis violacea DSM207
Thiohalocapsa halophila DSM6210
Sedimenticolaceae bacterium HK-STAS-PROT-129
Sedimenticolaceae bacterium 1371-Tw2
Lamprocystis purpurea DSM4197
Thiobaca trueperi DSM13587
Thiospirillum jenense DSM216
Thermochromatium vinosum DSM180
Thermochromatium tepidum ATCC 43061
Rhabdochromatium marinum DSM5261
Thiorhodovibrio winogradskyi 6511
Sedimenticolaceae bacterium Modern marine.mb.35
AAI (%)
100
80
60
40

Figure S9. Heatmap of the average Amino Acid Identity (AAI) estimated from pairwise genome comparisons of genomes used in the trees of the Figure 4 (*Azospirillaceae* family of the *Alphaproteobacteria*, CAIRSR01 order and *Chromatiaceae* family of the *Gammaproteobacteria*, respectively).

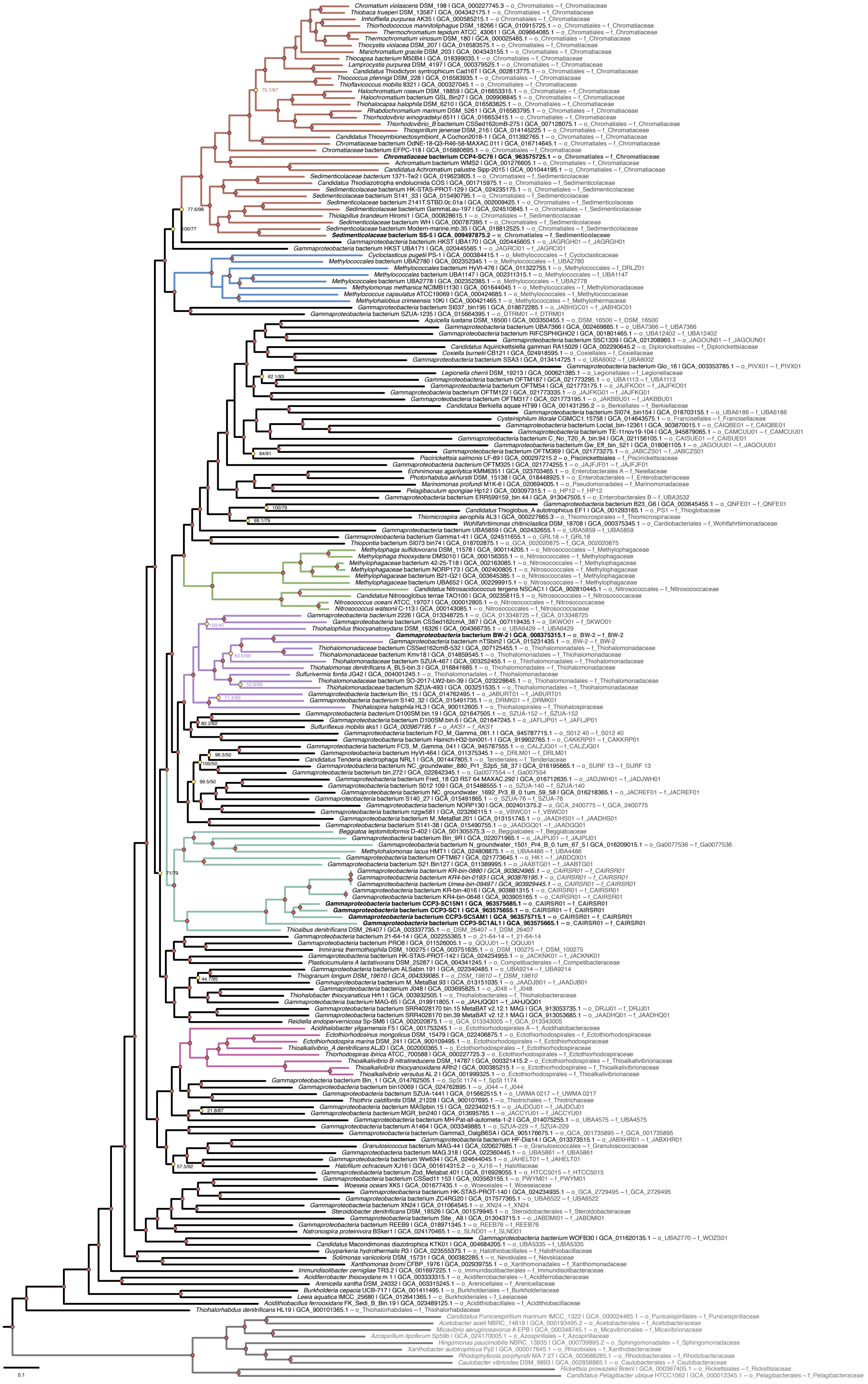

Figure S10. Maximum-likelihood tree of the *Gammaproteobacteria* class based on 120 conserved bacterial markers used for the GTDB classification showing the distribution of morphotype 3 and 4 in two different orders (*i.e.*, CAIRSR01 and *Chromatiales*, respectively). The tree was drawn as described in the Material and Methods section. We selected a set of genomes of good quality (*i.e.*, > 90% complete with < 5% redundancy according to CheckM v1.0.18 [2] (August 2023). The number of genomes meeting this criterion being very important (> 91k), we randomly selected a high-quality genome representing each order of the GTDB taxonomy [3], giving preference to representative genomes and genomes from type strains. For orders potentially close to $_{\text{iACC}}$MTB, such as *Thiohalomonadales*, *Methylococcales*, *Nitroscococcales* and *Chromatiales*, genomes from two genera for each family were selected. Finally, the dataset was completed with all the genomes identified as being in the same group than one or more $_{\text{iACC}}$MTB based on GTDB-tk [4] analysis. The tree was rooted with representative members of several *Alphaproteobacteria* orders (grey group) leading to a total of 211 genomes. Branch lengths represent the number of substitutions per site. The circles on the internal nodes represent the statistical support considered satisfactory when the likelihood rate (aLRT) is greater than 0.95 (estimated from 1000 replicas) and the non-parametric bootstrap value is greater than 80% (estimated from 500 replicas). The $_{\text{iACC}}$MTB genomes and that of the two MTB cultivated strains are shown in bold. A few colors are used to highlight certain important orders in our study. The corresponding Genbank accession numbers are given in the sequence names, along with the corresponding order name "o_" and family name "f_" in GTDB [3] (https://gtdb.ecogenomic.org). Two trees with a more reduced sampling were constructed from the clades in turquoise and in brown, positioning the $_{\text{iACC}}$MTB of morphotype 3 and 4 respectively (Fig. 4). A newick version is given in Data S1.

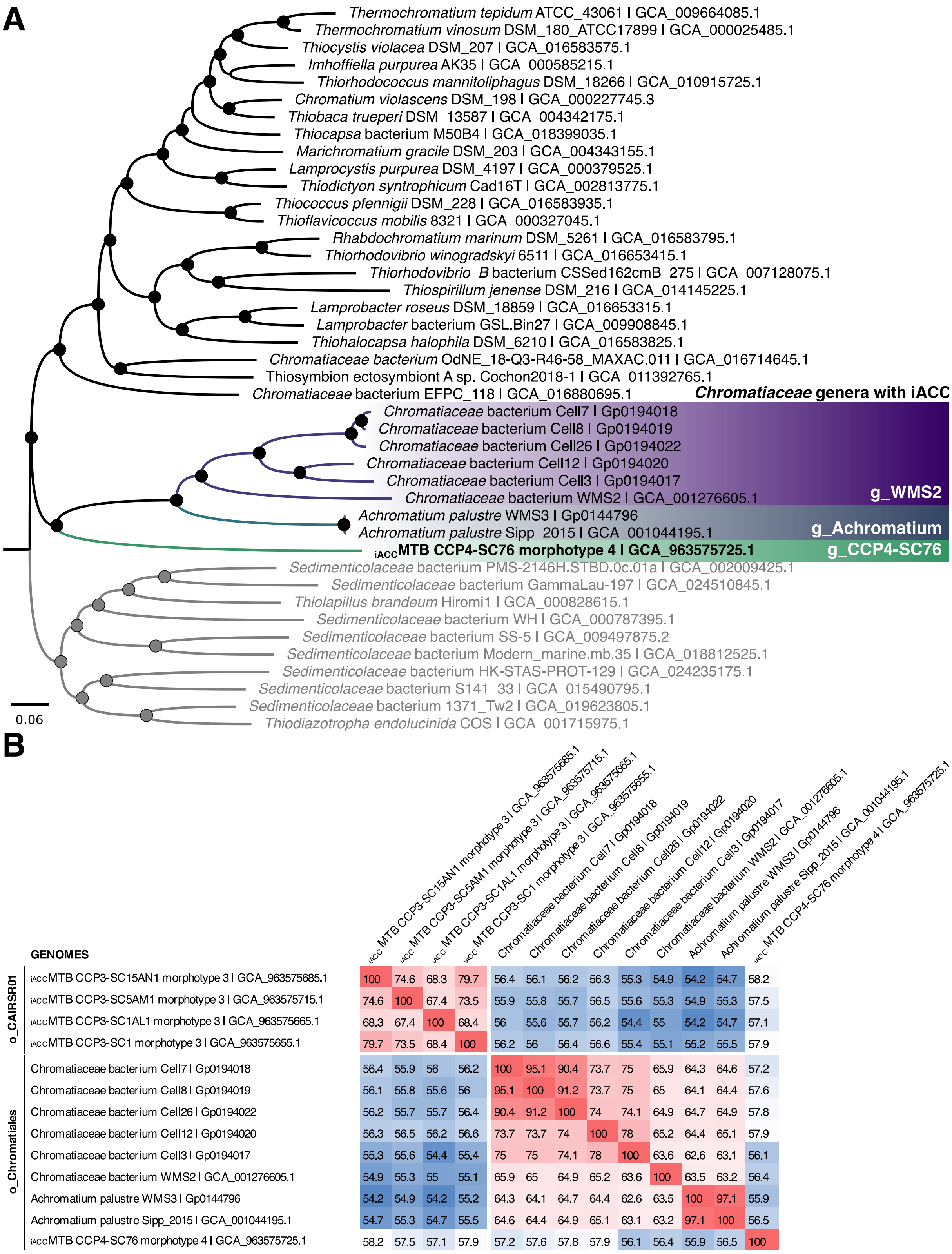

Figure S11. Maximum-likelihood tree of the *Chromatiaceae* family (A) and heatmap of AAI relatedness among the $_{iACC}$B genera including *Achromatium* related species (B). The tree was built as described in Figure 4 and includes additional freshwater high- and medium-quality MAGs related to *Achromatium* from Ionescu *et al.* [5]. The $_{iACC}$MTB of the morphotype 4 only forms a monophyletic group with *Achromatium* related species (Fig. S10). Here, we can

observe that freshwater $_{iACC}$B do not form a monophyletic group. According to the GTDB-tk classification and whole genome comparisons only: 1) the genus *Achromatium* would be represented by marine *Achromatium palustre* related species only, 2) the freshwater *Achromatium* species related to *Achromatium oxaliferum* should be reclassified in a new genus and 3), the $_{iACC}$MTB of the morphotype 4 represents a separated genus on their own. These results are further supported by the AAI relatedness. Indeed, AAI % values between *Achromatium* related species and magnetotactic *Gammaproteobacteria* forming iACC are below 65% which is in the range of threshold values defining new genera [6]. Although morphologically and functionally very similar, morphotype 3 and *Achromatium* do not belong to the same *Gammaproteobacteria* genus, family nor order.

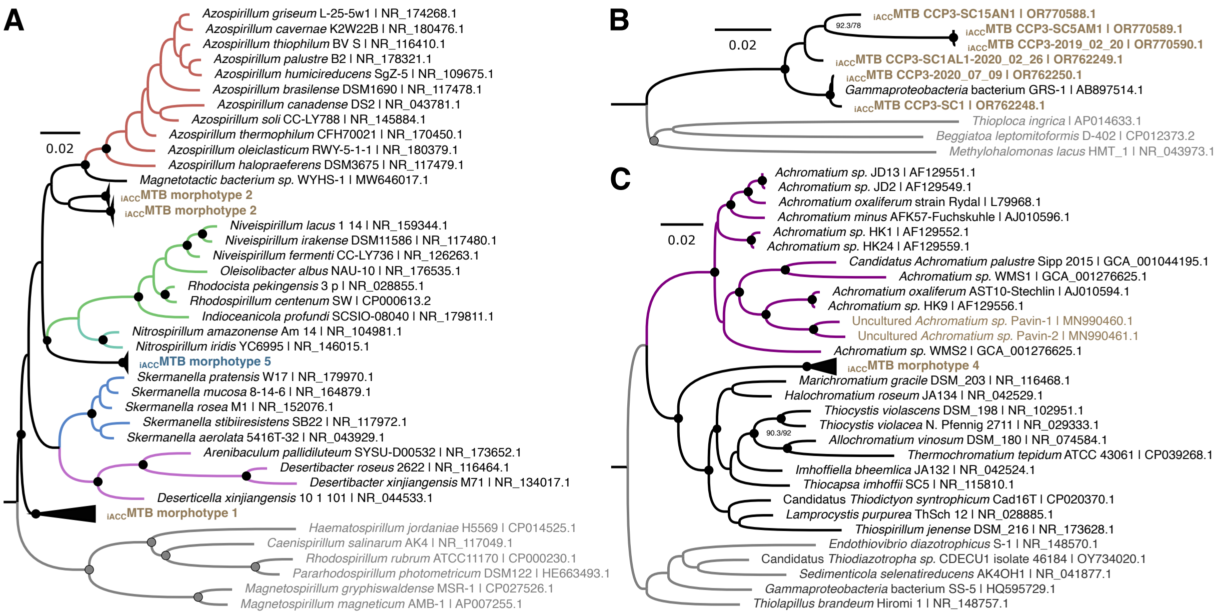

Figure S12. Phylogenetic trees based on the 16S rRNA gene sequence showing the relationships of $_{\text{iACC}}$MTB with other environmental bacteria. (**A**) Phylogenetic tree of the family *Azospirillaceae* (order *Rhodospirillales*, *Alphaproteobacteria*). (**B**) Phylogenetic tree of the genomic order CAIRSR01 of the *Gammaproteobacteria* class (GTDB taxonomy). (**C**) Phylogenetic tree of the family *Chromatiaceae* (order *Chromatiales*, *Gammaproteobacteria*). Sequences were retrieved from genomes, and from the NCBI database (https://www.ncbi.nlm.nih.gov). No sequence belonging to the same genus/species than those of $_{\text{iACC}}$MTB here were found in the SILVA SSU 138.2 database with the SINA ACT Tool (https://www.arb-silva.de/aligner; September 2024). Trees were constructed using the maximum likelihood method implemented in IQ-TREE [7]. The taxonomic samples used to root the 16S rRNA gene trees (in grey) are the same than those of the whole genome trees given in Fig. 4. $_{\text{iACC}}$MTB from the sediment are in brown whereas those of the water column are in blue. Branch lengths represent the number of substitutions per site. Circles on internal nodes represent statistical support considered satisfactory when the likelihood rate (aLRT) is greater than 0.95 (estimated from 1000 replicates) and the non-parametric bootstrap value is greater than 80% (estimated from 500 replicates). Trees topologies are overall not well supported. However, tree A shows that $_{\text{iACC}}$MTB do not cluster with the magnetotactic bacterium WYHS-1 forming calcium phosphates [8]. Tree B shows that the magnetotactic bacterium GRS-1 [9] belongs to the same *Gammaproteobacteria* species than the one identified in this study. Finally tree C shows that *Achromatium* species observed in Lake Pavin by Benzerara *et al.* [10] do not cluster with $_{\text{iACC}}$MTB of morphotype 4. The $_{\text{iACC}}$MTB sequences have been deposited in Genbank (OR539775 to OR539792; OR761886 to OR761890; OR762248 to OR762250;

OR761886 to OR761890; OR770588 to OR770590) and the corresponding accession numbers are given in the sequence names.

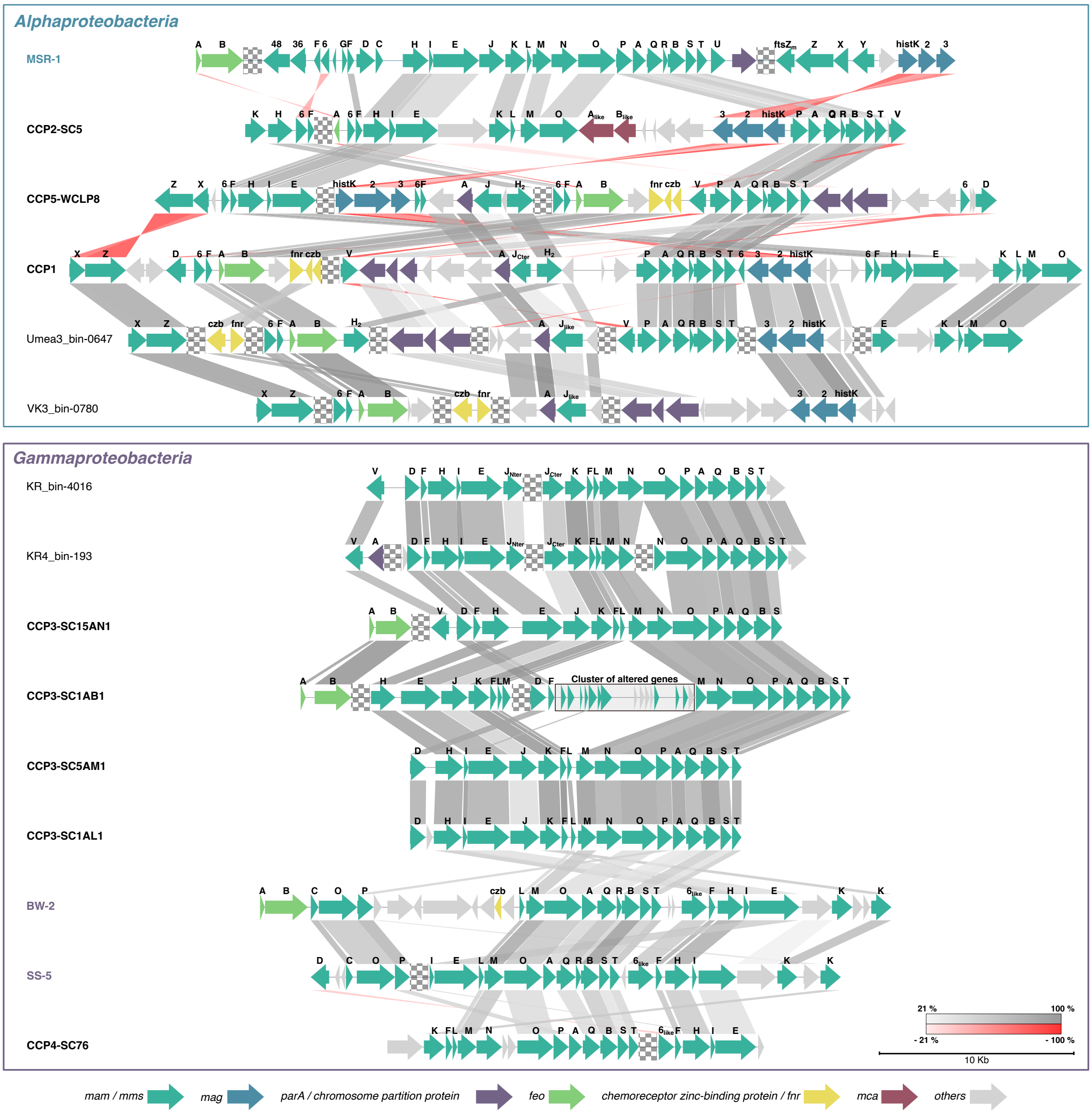

Figure S13. Conservation of magnetosome gene cluster (MGC) synteny of the iACCMTB sequenced in this study, the MAGs with which they cluster in phylogenetic trees and the magnetotactic *Pseudomonadota* model strains MSR-1, BW-2 and SS-5. Genomes are organized by class (*Alphaproteobacteria* and *Gammaproteobacteria*). Names in bold represent the iACCMTB genomes sequenced in this study: CCP-1 is the name given to the iACCMTB morphotype 1 genome; CCP2-SC5 to morphotype 2; CCP5-WCLP8 to water column morphotype 5; CCP3-SC1AL1, CCP3-SC5AM1, CCP3-SC1AB1, CCP3-SC15AN1 to morphotype 3; and CCP4-SC76 to morphotype 4. Names in regular font represent MAG from stratified environments obtained by Buck *et al.* [11]. Each arrow represents a gene of a color corresponding to a specific operon in MSR-1 [12]. Grey genes are genes of unknown function or not conserved in MTB. Checkerboards represent mainly truncations and sometimes regions spacing two operons. Sequence identities between Reciprocal Best Hits (RBH) were estimated

with MMseqs2 [13] and are represented by bands, with their intensity reflecting the percentage of identity. Some homologs are not linked to due to high sequence divergence and/or the presence of multiple paralogs. Homologues families were then determined by the presence of conserved domains using the Microscope platform [14].

2. Tables

Table S1. List of $_{iACC}$MTB genomes used in this study
See the Supplementary Table file: Table S1 (XLSX).

Table S2. Comparative analysis of metabolic pathways predicted in at least in one of the $_{iACC}$MTB genomes based on the MetaCyc pathways database
See the Supplementary Table file: Table S2 (XLSX).

3. Videos

Video S1. Light microscope observation of a cell pellet magnetically concentrated from the sediments of Lake Pavin
See the Supplementary Data file: Video S1 (MP4).

4. Data

Data S1. Maximum-likelihood tree of the Gammaproteobacteria class
See the Supplementary Data file: Data S1 (TXT).

5. References

1. Monteil CL, Benzerara K, Menguy N, Bidaud CC, Michot-Achdjian E, Bolzoni R, et al. Intracellular amorphous Ca-carbonate and magnetite biomineralization by a magnetotactic bacterium affiliated to the *Alphaproteobacteria*. *ISME J* 2021; **15**: 1–18.

2. Parks DH, Imelfort M, Skennerton CT, Hugenholtz P, Tyson GW. CheckM: assessing the quality of microbial genomes recovered from isolates, single cells, and metagenomes. *Genome Res* 2015; **25**: 1043–1055.

3. Parks DH, Chuvochina M, Rinke C, Mussig AJ, Chaumeil P-A, Hugenholtz P. GTDB: an ongoing census of bacterial and archaeal diversity through a phylogenetically consistent, rank normalized and complete genome-based taxonomy. *Nucleic Acids Res* 2022; **50**: D785–D794.

4. Chaumeil P-A, Mussig AJ, Hugenholtz P, Parks DH. GTDB-Tk: a toolkit to classify genomes with the Genome Taxonomy Database. *Bioinformatics* 2020; **36**: 1925–1927.

5. Ionescu D, Bizic-Ionescu M, De Maio N, Cypionka H, Grossart H-P. Community-like

genome in single cells of the sulfur bacterium *Achromatium oxaliferum*. *Nat Commun* 2017; **8**: 455.

6. Konstantinidis KT, Tiedje JM. Genomic insights that advance the species definition for prokaryotes. *Proc Natl Acad Sci* 2005; **102**: 2567–2572.

7. Minh BQ, Schmidt HA, Chernomor O, Schrempf D, Woodhams MD, von Haeseler A, et al. IQ-TREE 2: new models and efficient methods for phylogenetic inference in the genomic era. *Mol Biol Evol* 2020; **37**: 1530–1534.

8. Liu P, Liu Y, Ren X, Zhang Z, Zhao X, Roberts AP, et al. A novel magnetotactic alphaproteobacterium producing intracellular magnetite and calcium-bearing minerals. *Appl Environ Microbiol* 2021; **87**: e0155621.

9. Taoka A, Kondo J, Oestreicher Z, Fukumori Y. Characterization of uncultured giant rod-shaped magnetotactic *Gammaproteobacteria* from a freshwater pond in Kanazawa, Japan. *Microbiol Read Engl* 2014; **160**: 2226–2234.

10. Benzerara K, Bolzoni R, Monteil C, Beyssac O, Forni O, Alonso B, et al. The gammaproteobacterium *Achromatium* forms intracellular amorphous calcium carbonate and not (crystalline) calcite. *Geobiology* 2021; **19**: 199–213.

11. Buck M, Garcia SL, Fernandez L, Martin G, Martinez-Rodriguez GA, Saarenheimo J, et al. Comprehensive dataset of shotgun metagenomes from oxygen stratified freshwater lakes and ponds. *Sci Data* 2021; **8**: 131.

12. Uebe R, Schüler D. Magnetosome biogenesis in magnetotactic bacteria. *Nat Rev Microbiol* 2016; **14**: 621–637.

13. Steinegger M, Söding J. MMseqs2 enables sensitive protein sequence searching for the analysis of massive data sets. *Nat Biotechnol* 2017; **35**: 1026–1028.

14. Vallenet D, Calteau A, Dubois M, Amours P, Bazin A, Beuvin M, et al. MicroScope: an integrated platform for the annotation and exploration of microbial gene functions through genomic, pangenomic and metabolic comparative analysis. *Nucleic Acids Res* 2020; **48**: D579–D589.
