# Supplementary material for: Magnetotactic bacteria affiliated with diverse Pseudomonadota families biomineralize intracellular Ca-carbonate
Source: ISME J. 2025 Jan 7;19(1):wrae260. doi: 10.1093/ismejo/wrae260 (PMC11773610; doi:10.1093/ismejo/wrae260)
Supplement: 20241219_Supplementary_Notes_wrae260 [file 20241219_supplementary_notes_wrae260.pdf]

Supplementary Notes

Magnetotactic bacteria affiliated with diverse *Pseudomonadota* families biomineralize intracellular Ca-carbonate

Camille C. Mangin[1], Karim Benzerara[2], Marine Bergot[1], Nicolas Menguy[2], Béatrice Alonso[1], Stéphanie Fouteau[3], Raphaël Méheust[3], Daniel Chevrier[1], Christian Godon[1], Elsa Turrini[1], Neha Mehta[2], Arnaud Duverger[2], Cynthia Travert[2], Vincent Busigny[4], Elodie Duprat[2], Romain Bolzoni[1,2], Corinne Cruaud[5], Eric Viollier[6], Didier Jézéquel[4,7], David Vallenet[3], Christopher T. Lefèvre[1] & Caroline L. Monteil[1*]

[1] *Université Aix-Marseille, CNRS, CEA, UMR7265 Institut de Biosciences and Biotechnologies d'Aix-Marseille, CEA Cadarache, F-13108 Saint-Paul-lez-Durance, France.* [2] *Sorbonne Université, Institut de Minéralogie, de Physique des Matériaux et de Cosmochimie (IMPMC) - UMR 7590 CNRS MNHN - 4, place Jussieu - BC 115 - 75252 Paris Cedex 5.* [3] *LABGeM, Génomique Métabolique, CEA, Genoscope, Institut François Jacob, CNRS, Université d'Évry, Université Paris-Saclay, Evry, France.* [4] *Université Paris Cité, Institut de Physique du Globe de Paris, CNRS, F-75005, Paris, France.* [5] *Genoscope, Institut de biologie François Jacob, CEA, Université Paris-Saclay, Evry, France.* [6] *LSCE, CEA/CNRS/UVSQ/IPSL, Université Paris Saclay, Université Paris Cité, France 91191 Gif-sur-Yvette Cedex.* [7] *UMR CARRTEL, INRAE-USMB, Thonon, France*

*Corresponding author: Caroline L. Monteil, Université Aix-Marseille, CNRS, CEA, UMR7265 Institut de Biosciences and Biotechnologies d'Aix-Marseille, CEA Cadarache, F-13108 Saint-Paul-lez-Durance, France.
Email: caroline.monteil@cea.fr

Contents

1. **Extended Methods**

   Method S1. Analysis of MTB populations in the water column of the Lake Pavin

   Method S2. Estimation of $_{iACC}$MTB morphotypes relative frequency in shallow sediments

   Method S3. Additional information regarding the fluorescence *in situ* hybridization (FISH) experiment

   Method S4. Protocol for ultra-thin sections preparation

   Method S5. Genome sequencing

2. **References**

1. Extended Methods

Method S1. Analysis of MTB populations in the water column of Lake Pavin

According to previous studies [1, 2], a MTB population forming iACC inhabits the Lake Pavin water column just below the oxic-anoxic transition zone. To further get insight into the diversity in the water column, we led a field campaign during October 2021, the 26-29th. In line with previous studies, an online pumping system previously developed [3] was used to sample water between 45 and 65 m. This technique allows sampling along a vertical transect with a 10 cm resolution from a platform near the center of Lake Pavin (45.495792°N, 2.888117°E) (Method S1 figure). During previous profiling campaigns, the maximum MTB abundance was systematically detected below the O_2 detection limit in a specific conductivity range, and close to the maximum redox potential decay and turbidity peak [3]. The oxic-anoxic transition zone fluctuating over seasons and years, an initial geochemical profiling was thus performed to roughly localize this zone, then a second more resolved profile was carried out around the peak of abundance. Once the depth of the $_{iACC}$MTB abundance peak identified, several samples between 1 L and 200 L were collected for $_{iACC}$MTB characterization and DNA extraction.

The depth profiles of dissolved oxygen, conductivity and redox potential (ORP) were measured *in situ* in the water column using a YSI EXO2 CTD probe. The oxygen sensor was calibrated against ambient air saturated with water vapor (local O_2 saturation of 100%), and "zero" O_2 saturation was checked using a 10% w/v sodium sulfite solution. Specific conductivity at 25°C was calibrated against a standard KCl solution (1.413 $\mu S.cm^{-1}$) and pH using 3 buffers (4/7/10). The various probes were attached to the end of the sampling tube, so that these parameters can be monitored directly during sampling. Once the oxic-anoxic transition zone localized, 1-L bottles were filled to their maximum capacity every 10 cm over 2 meters below O_2 disappearance, taking great care to not over-oxygenate the samples. A classic magnetic concentration was then performed, a maximum 1 hour after sampling, as previously described [2]. Magnetically concentrated cells were observed using a Zeiss Primo Star optical microscope. The different morphologies and magnetotactic behaviors were distinguished using the 40 and 63 objectives.

Similar distribution patterns were observed for magnetotactic cocci (MTBc) and $_{iACC}$MTB concentrations to those of a previous study [2] (Method S1 figure). The MTBc maximum abundance (i.e., 1.5×10^4 cells/mL) was localized around 52 m whereas that of $_{iACC}$MTB (i.e., 2×10^3 cells/mL) was localized 3 m deeper. Several samples were collected to prepare microscopy grids at the Besse scientific station 10 min by car, while some of them were kept at 4°C for further molecular work. MTB populations quickly disappeared several

days later as this type of mesocosm does not preserve chemical gradients. Finally, 200 L of water were pumped at 55 m every 3 h for 16 h long and magnetically concentrated in 20-L aquaria. Collected pellets were pulled to get a final pellet of 10^9–10^{10} cells, stored at –20°C before DNA extraction.

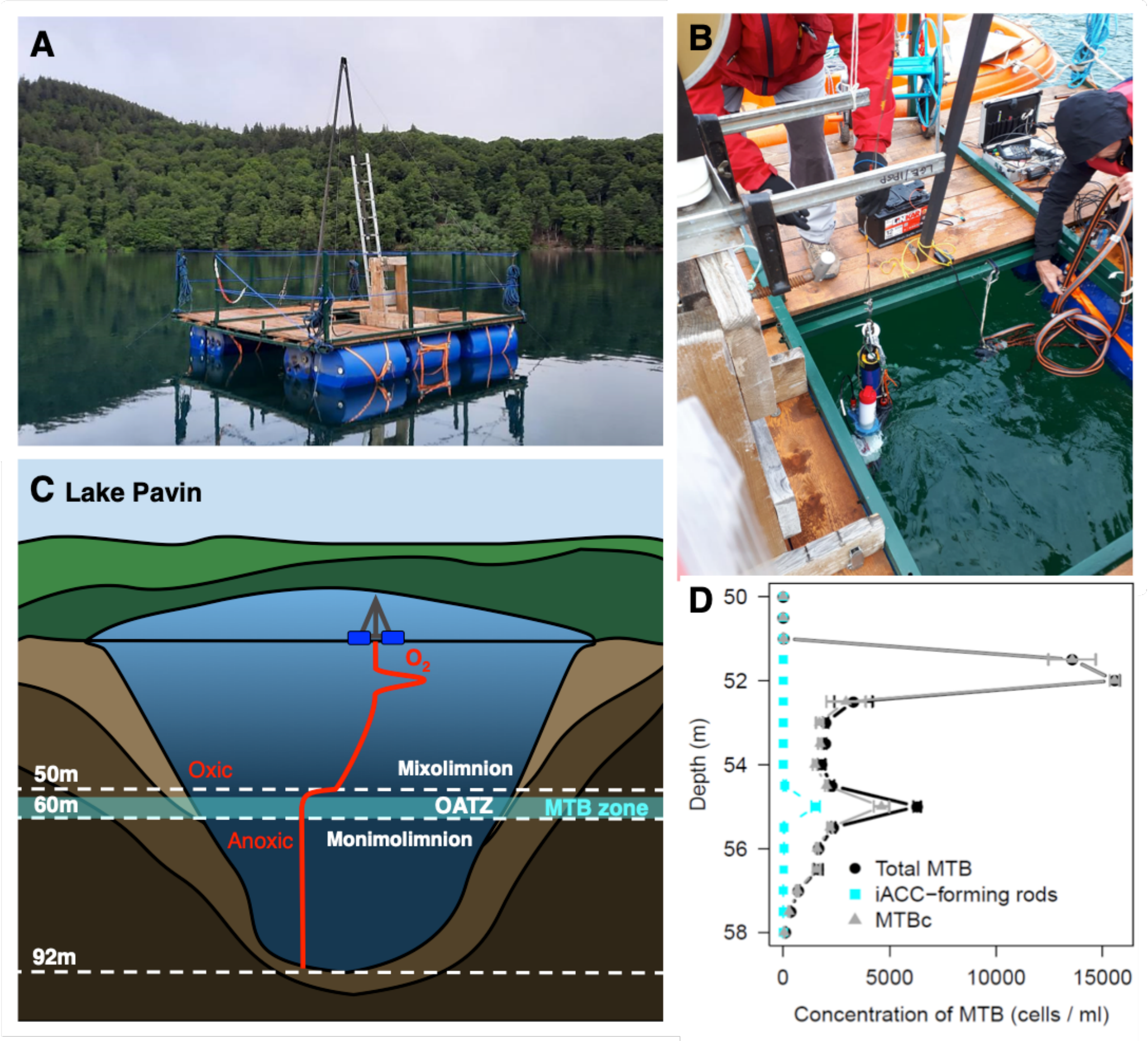

Method S1 figure. Analysis of MTB populations in the water column of Lake Pavin. (**A**) Image of the platform positioned at the center of the lake from where water column samples were collected, and geochemical profiling was done. (**B**) Image of the platform center showing the online pumping system with a pipe immersed in the entire water column of the lake and probes to perform the geochemical profiling. (**C**) Schematic representation of the chemical stratification of the lake showing the zone just below the oxic-anoxic transition zone where MTB are localized and (D) their distribution over 8 m in October 2021. Error bars represent standard errors (n=3).

Method S2. Estimation of $_{iACC}$MTB morphotypes relative frequency in shallow sediments

The way sediment samples are usually collected to seek for MTB may present biases for getting a quantitative estimation of each population compared to the others. We thus applied a small sediment coring approach to estimate more precisely the relative abundance of $_{iACC}$MTB populations in shallow sediments, which ensures reproducibility. A 50-mL syringe opened at both edges was used to core sediment over 4 cm below the water-sediment interface, which represents a known volume of 21 cm^3 of sediment (Method S2 figure). To do so, the syringe was pressed progressively into the sediment while rotating and pulling the piston; excess sediment collected (> 4 cm depth) was discarded (Method S2 figure). Cores were collected at each sampling site LP1, LP2, LP3 in triplicate. Cores were then transferred from the syringe to a 50 mL Falcon tube and supplemented with surface water from the sampled site filtered with a 0.22-µm Nitrocellulose filter. The same magnetic sorting protocol described in the Material and Methods section was then applied, with the exception that a small neodymium iron-boron magnet was taped to the surface of the Falcon tube to facilitate holding the magnet during concentration. After 2h of magnetic sorting, 100 µL of the bacterial pellet located against the magnet was collected and transferred to a 1.5 mL Eppendorf tube. The cells were homogenized with gentle back-and-forth movements using a micropipette. A volume of 50 µL of this tube was then removed and transferred to a microscope coverslip and observed under the MO using the hanging drop technique. A 10-minutes waiting period was required for the cells to migrate to the edge of the drop and be counted.

Three successive magnetic concentrations and counts were carried out from the same core/falcon tube to estimate a depletion factor of each $_{iACC}$MTB population between each magnetic concentration. Using this approach, it was possible to obtain (i) a cell concentration (cells / mL) and (ii) an estimate of the total number of each cell population contained in the Falcon tube. The number of MTB cells counted is then converted into relative frequency, given by the ratio (total number of $_{iACC}$MTB morphotype X) / (total number of $_{iACC}$MTB) to obtain the relative abundance of certain MTB with varied morphologies compared to others.

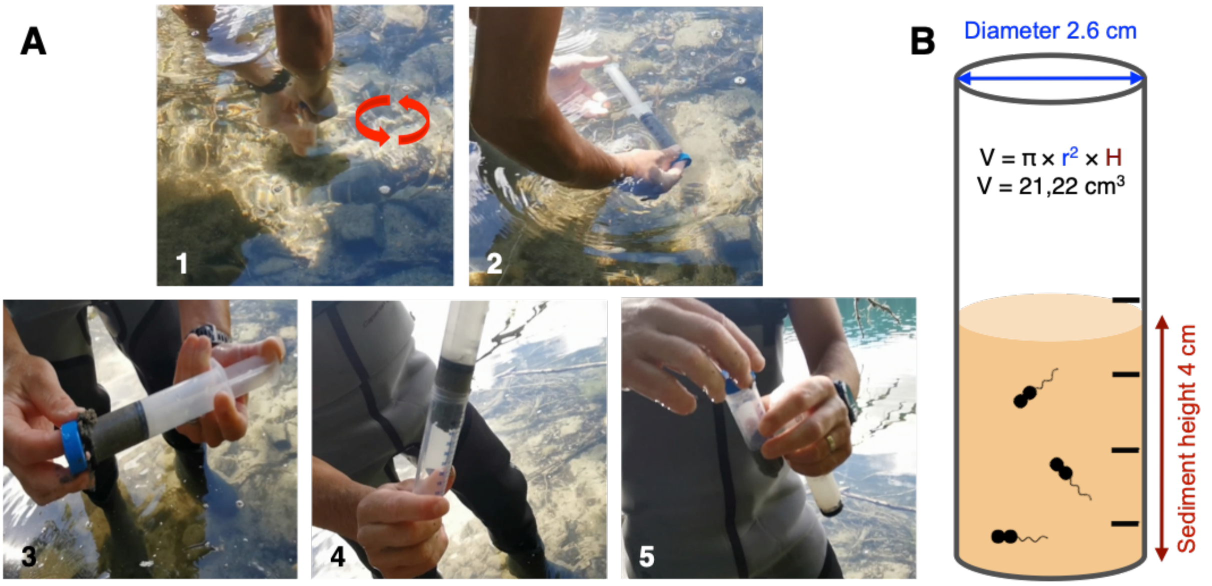

Method S2 figure. Sediment coring to estimate $_{iACC}$MTB morphotypes relative frequency in shallow sediments. (**A**) Images illustrating the 5 steps of coring using a 50-mL syringe, cut at its extremity, to maintain sediment and gradients integrity. (**B**) Schematic representation of the sampled sediment core.

Method S3. Additional information regarding the fluorescence *in situ* hybridization (FISH) experiment

Specific probes of the 16S rRNA gene sequence of each species or group of species were designed for each morphotype. The absence of specificity with other species was verified by alignment (BLASTN, default parameters) with the most similar 16S rRNA gene sequences found in the GenBank database in December 2021. Probe specificity was assessed using the PROBE_MATCH program in RDP-II [4]. The closest non-targeted sequences contain at least one mismatch with the specific probes. The fluorochrome ATTO488 was added on the 5' side of these nucleotide probes (Method S3 table) and were ordered from Eurofins Genomics (Ebersberg, Germany).

Slide preparations for confocal microscopy observations were realized with $_{iACC}$MTB and other MTB used as negative controls. A clean 10-µL drop of filtered Lake Pavin water (0.2 µm) and a 20-µL drop of magnetically concentrated cells were deposited on a Superfrost Plus Gold adhesive slide (THERMO SCIENTIFIC MENZEL) with the south pole of a magnet positioned on the side of the slide with the filtered water to guide MTB towards the edge free of dirt from the sample. After 10 min the part of the drop containing sediment particles is gently removed. Cells adsorbed on the slide were fixed overnight in 4% paraformaldehyde solution. The fixed cells were then dehydrated by serial incubations in 50%, 80% and 100% ethanol for 5 min each. The hybridization solution contained 10 ng / mL of probe, 0.9 M NaCl, 20 mM Tris-HCl (pH 7.4), 1 mM Na_2EDTA, and 0.01% sodium dodecyl sulfate (SDS), using the recommended hybridization and wash stringencies for each probe (35% for specific probes). Hybridization was performed at 46°C for 2 h. The EUB388 probe with the fluorochrome ATTO633 added on its 5’ side was used to selectively mark the presence of bacterial DNA [5]. Slides were also stained with 4,6-diamidino-2-phenyl indole (DAPI) contained in the mounting solution (ProLong with DAPI, ThermoFisher Scientific) added to the slide. Slides were stored at 4°C in a humidity chamber before observation. Hybridization assays were analyzed using a Zeiss LSM980 confocal fluorescence microscope (BIAM, France). The probes CCP2p, CCP3p or CCP4p (Method S3 table), and therefore the taxonomic affiliation, were validated only when cells of the morphotype analyzed fluoresce at the wavelengths of the fluorochrome ATTO488 (500-520 nm), whereas other MTB cells fluoresce at the wavelengths of the fluorochromes DAPI (350-470 nm) and ATTO633 (630-651 nm) only. Images were collected with ZEN software.

Method S3 table. Oligonucleotide probes developed for FISH experiments using the 16S rRNA sequences of morphotypes 2, 3 and 4 (named CCP2p, CCP3p and CCP4p,

respectively). Each oligonucleotide probe is associated with a fluorochrome and its nucleotide position is indicated. Between 4 and 9 sequences obtained from single-cell were used to build the probe. The probe EUB338 is specific to *Eubacteria*.

**Probe name**	**Sequence 5’-> 3’**	**Position**	**Publication**
EUB338	5'-ATTO633-GCTGCCTCCCGTAGGAGT-3'	338-355	Amaan *et al.* (1990)
CCP2p	5’-ATTO488-CCATCCCATGGCGATAAATC-3’	175-194	This study
CCP3p	5’-ATTO488-GAAGGCACTCTCATGTCTCCATAAG-3’	992-1016	This study
CCP4p	5’-ATTO488-CTAGCTAATCGGAC GTGGGCTCATCTGATAG-3’	201-237	This study

Method S4. Protocol for ultrathin sections preparation

MTB were harvested from the microcosms as described above. After collecting several pellets of magnetically concentrated cells, they were fixed in a solution of 2.5% Glutaraldehyde and 0.1 M Cacodylate at pH 7 prepared in filtered Lake Pavin water, then stored at 4°C. Osmium tetroxide 1% was used to contrast the samples for electron microscope observations. After incubation for 1 h at 4°C, the samples were washed, mixed with 2% "low melting" agarose (Sigma-Aldrich) and cut into small blocks of approximately 1 mm^3. The blocks were placed in tubes containing Uranyless, then stored at 4°C. Samples were progressively dehydrated in alcohol baths at different concentrations (30, 50, 70, 90% EtOH). They were then progressively embedded in a specific resin composed of Embed 812, DDSA, NMA and BDMA. The inclusions were transferred to molds and incubated at 60°C for 48 hours to allow the resin to solidify. The resin blocks were cut in several stages to obtain thin sections 100 nm thick. Thin sections were made using diamond knives on the Leica EM UC7 Ultramicrotome. The thin sections were placed on electron microscopy grids, then stained with Uranyless and 3% lead citrate (Reynolds Lead Citrate, Uranyless).

Method S5. Genome sequencing

First, 3 µg of amplified DNA were treated with T7 Endonuclease I (New England Biolabs) to resolve branches due to the multiple displacement mechanism. For Illumina sequencing, 50 to 250 ng DNA were sonicated to a 100–1,000 bp size range using the E220 Covaris instrument (Covaris, Inc.). The fragments were end repaired and 3′ adenylated, and NEXTflex HT barcodes were added (BioScientific Inc.). The ligated products were amplified using 12 PCR cycles with the Hifi HotStart ReadyMix kit (Kapa Biosystems) and purified with 0.8 × AMPure XP reagent. After library profile analysis using the Agilent 2100 Bioanalyzer and quantitative PCR (qPCR) (MxPro; Agilent Technologies), the library was sequenced on a MiSeq or NovaSeq 6000 instrument (Illumina). The short reads were trimmed by removing low-quality ($Q<20$) nucleotides, sequencing adaptors, and sequences of <30 nucleotides (nt), using the FastX-Toolkit package to obtain between 3 and 8 million of paired reads per genome.

For long-read sequencing, 1 µg DNA was used for the library preparation following the 1D Native barcoding genomic DNA protocol with the EXP-NBD104 and SQK-LSK109 ligation kit (Oxford Nanopore). The library was sequenced using a Nanopore R9.4.1 revD flow cell, the PromethION device and the MinKNOW user interface software (v4.0.5 for samples CCP3-SC1, CCP2-SC5; v5.1.0 for samples CCP3-SC15, CCP3-SC1AL1, CCP3-SC5AM1). The basecalling was performed using Guppy (v4.0.11+f1071ce for samples CCP3-SC1, CCP2-

SC5; v6.1.5+446c35524 for samples CCP3-SC15, CCP3-SC1AL1, CCP3-SC5AM1). Between 18 k and 1 million of cleaned sequences were used for each genome.

For the metagenome sequencing, total DNA was extracted with a conventional phenol/chloroform method as [6], directly from homogenates using TE buffer supplemented with proteinase K (20 mg/mL), lysozyme (10 mg/mL), and SDS (10%). Samples were treated with phenol/chloroform/isoamyl alcohol (25:24:1, pH 8), briefly mixed and centrifuged for 1 h at 4°C. The supernatant was then transferred to sterile tubes filled with chloroform/isoamyl alcohol (24:1), mixed and centrifuged for 1 min at 4°C. The aqueous phase was then collected. DNA was precipitated for one hour with absolute isopropanol at room temperature, pelleted by centrifugation (10 000 rpm for 5 min at 4°C), washed in 70% ethanol and eluted in 50 µL of TE buffer. An equivalent sequencing procedure was performed following the same methods than SAGs but generating ~ 148 million paired Illumina reads.

2. References

1. Monteil CL, Benzerara K, Menguy N, Bidaud CC, Michot-Achdjian E, Bolzoni R, et al. Intracellular amorphous Ca-carbonate and magnetite biomineralization by a magnetotactic bacterium affiliated to the Alphaproteobacteria. *ISME J* 2021; 15: 1–18.
2. Bidaud CC, Monteil CL, Menguy N, Busigny V, Jézéquel D, Viollier É, et al. Biogeochemical Niche of Magnetotactic Cocci Capable of Sequestering Large Polyphosphate Inclusions in the Anoxic Layer of the Lake Pavin Water Column. *Front Microbiol* 2022; 0.
3. Busigny V, Mathon FP, Jézéquel D, Bidaud CC, Viollier E, Bardoux G, et al. Mass collection of magnetotactic bacteria from the permanently stratified ferruginous Lake Pavin, France. *Environ Microbiol* 2021.
4. Cole JR, Chai B, Marsh TL, Farris RJ, Wang Q, Kulam SA, et al. The Ribosomal Database Project (RDP-II): previewing a new autoaligner that allows regular updates and the new prokaryotic taxonomy. *Nucleic Acids Res* 2003; 31: 442–443.
5. Amann RI, Krumholz L, Stahl DA. Fluorescent-oligonucleotide probing of whole cells for determinative, phylogenetic, and environmental studies in microbiology. *J Bacteriol* 1990; 172: 762–770.
6. Benidire L, El Khalloufi F, Oufdou K, Barakat M, Tulumello J, Ortet P, et al. Phytobeneficial bacteria improve saline stress tolerance in *Vicia faba* and modulate microbial interaction network. *Sci Total Environ* 2020; 729: 139020.
